# Tailored vs. General COVID-19 prevention for adults with mental disabilities residing in group homes: a randomized controlled effectiveness-implementation trial

**DOI:** 10.1186/s12889-024-18835-w

**Published:** 2024-06-26

**Authors:** Stephen Bartels, Julie H. Levison, Hao D. Trieu, Anna Wilson, David Krane, David Cheng, Haiyi Xie, Karen Donelan, Bruce Bird, Kim Shellenberger, Elizabeth Cella, Nicolas M. Oreskovic, Kelly Irwin, Kelly Aschbrenner, Ahmed Fathi, Stefanie Gamse, Sibyl Holland, Jessica Wolfe, Cindy Chau, Adeola Adejinmi, Jasmine Langlois, Jean-Louise Reichman, Lisa I. Iezzoni, Brian G. Skotko

**Affiliations:** 1 Mongan Institute, Massachusetts General Hospital,, 100 Cambridge St., Suite 1600, Boston, MA 02114 USA; 2 Department of Medicine, Harvard Medical School, Massachusetts General Hospital, 55 Fruit St., Gray 7-730, Boston, MA 02114 USA; 3 Department of Biostatistics, Harvard Medical School, Massachusetts General Hospital, 50 Staniford Street, Suite 560, Boston, MA 02114 USA; 4 Department of Biomedical Data Science, Geisel School of Medicine at Dartmouth, Williamson Translational Research Building, Third Floor, HB 7261, 1 Medical Center Drive, Lebanon, NH 03756 USA; 5 Department of Psychiatry, Geisel School of Medicine at Dartmouth, Dartmouth-Hitchcock Medical Center, One Medical Center Drive, Lebanon, NH 03756 USA; 6 Down Syndrome Program, Division of Medical Genetics and Metabolism, Department of Pediatrics, Massachusetts General Hospital, 125 Nashua Street, Suite 821, Boston, MA 02114 USA; 7 Department of Psychiatry, Harvard Medical School, Massachusetts General Hospital, 55 Fruit Street, Boston, MA 02114 USA; 8 Vinfen Corporation, 950 Cambridge Street, Cambridge, MA 02141 USA; 9 Bay Cove Human Services, 66 Canal Street, Boston, MA 02114 USA; 10 Advocates, Inc., 1881 Worcester Rd., Framingham, MA 01701 USA; 11 North Suffolk Mental Health Association, 301 Broadway, Chelsea, MA 02150 USA; 12 Department of Pediatrics, Harvard Medical School, Massachusetts General Hospital, 55 Fruit Street, Boston, MA 02214 USA

**Keywords:** COVID-19 prevention, Congregate care settings, Serious mental illness, Intellectual and developmental disability, Mental disabilities, Vaccine hesitancy, Vaccine acceptance, Health disparities, Intersectionality, Equity-focused implementation, Hybrid effectiveness-implementation trial

## Abstract

**Background:**

People with serious mental illness (SMI) and people with intellectual disabilities/developmental disabilities (ID/DD) are at higher risk for COVID-19 and more severe outcomes. We compare a tailored versus general best practice COVID-19 prevention program in group homes (GHs) for people with SMI or ID/DD in Massachusetts (MA).

**Methods:**

A hybrid effectiveness-implementation cluster randomized control trial compared a four-component implementation strategy (*Tailored Best Practices*: TBP) to dissemination of standard prevention guidelines (*General Best-Practices*: GBP) in GHs across six MA behavioral health agencies. GBP consisted of standard best practices for preventing COVID-19. TBP included GBP plus four components including: (1) trusted-messenger peer testimonials on benefits of vaccination; (2) motivational interviewing; (3) interactive education on preventive practices; and (4) fidelity feedback dashboards for GHs. Primary implementation outcomes were full COVID-19 vaccination rates (baseline: 1/1/2021–3/31/2021) and fidelity scores (baseline: 5/1/21–7/30/21), at 3-month intervals to 15-month follow-up until October 2022. The primary effectiveness outcome was COVID-19 infection (baseline: 1/1/2021–3/31/2021), measured every 3 months to 15-month follow-up. Cumulative incidence of vaccinations were estimated using Kaplan-Meier curves. Cox frailty models evaluate differences in vaccination uptake and secondary outcomes. Linear mixed models (LMMs) and Poisson generalized linear mixed models (GLMMs) were used to evaluate differences in fidelity scores and incidence of COVID-19 infections.

**Results:**

GHs (*n*=415) were randomized to TBP (*n*=208) and GBP (*n*=207) including 3,836 residents (1,041 ID/DD; 2,795 SMI) and 5,538 staff. No differences were found in fidelity scores or COVID-19 incidence rates between TBP and GBP, however TBP had greater acceptability, appropriateness, and feasibility. No overall differences in vaccination rates were found between TBP and GBP. However, among unvaccinated group home residents with mental disabilities, non-White residents achieved full vaccination status at double the rate for TBP (28.6%) compared to GBP (14.4%) at 15 months. Additionally, the impact of TBP on vaccine uptake was over two-times greater for non-White residents compared to non-Hispanic White residents (ratio of HR for TBP between non-White and non-Hispanic White: 2.28, *p* = 0.03).

**Conclusion:**

Tailored COVID-19 prevention strategies are beneficial as a feasible and acceptable implementation strategy with the potential to reduce disparities in vaccine acceptance among the subgroup of non-White individuals with mental disabilities.

**Trial registration:**

ClinicalTrials.gov, NCT04726371, 27/01/2021. https://clinicaltrials.gov/study/NCT04726371.

**Supplementary Information:**

The online version contains supplementary material available at 10.1186/s12889-024-18835-w.

## Introduction

People with mental disabilities including serious mental illness (SMI) and intellectual disabilities/developmental disabilities (ID/DD) are at disproportionately high risk for COVID-19 infections and poor outcomes, including hospitalization and death, due to comorbid health conditions [1–7]. Adults with SMI and ID/DD have high rates of smoking, obesity, chronic obstructive pulmonary disease, cardiovascular disease, and diabetes [8–16]. Many individuals with SMI and ID/DD also have cognitive, psychological, behavioral, and physical challenges contributing to reduced adoption of infection preventive practices such as use of masks and vaccine acceptance [2, 5, 7]. Furthermore, congregate care settings in which many people with SMI and ID/DD live carry many of the same higher risks of COVID-19 transmission affecting assisted-living settings and nursing homes across the nation [17–20].

Despite payment reforms and mandated best practices for COVID-19 for congregate care settings [1, 21], rates of COVID-19 early in the pandemic were 8 times higher (12.0%) for residents with SMI and ID/DD and 2 times higher for staff (3.0%), compared to the general population in surrounding “hotspot” communities (1.5%) [22–24]. Despite reduction in severity of illness associated with COVID-19 vaccination, vaccine hesitancy remains a major impediment to uptake, especially among populations with disabilities and among racially and ethnically diverse individuals [25–32]. Little is known about optimal strategies for effectively achieving vaccine uptake and related COVID-19 prevention practices in ethnically diverse individuals with mental disabilities.

The overall aim of this randomized effectiveness-implementation trial is to compare the effectiveness of an implementation strategy specifically tailored for a racially and ethnically diverse group of residents and staff in GHs for adults with SMI and ID/DD to standard dissemination of best practices to prevent COVID-19 and adverse outcomes. The tailored implementation strategy components consist of trusted**-**messenger peer testimonials on benefits of vaccination; motivational interviewing; interactive education, and GH fidelity feedback dashboards. In this randomized trial, we compared “Tailored Best Practice” (TBP) implementation strategy to “General Best Practices” (GBP) with respect to effectiveness and implementation outcomes for evidence-based COVID-19 prevention practices including screening, vaccination, face mask use, and hand washing [33].

## Methods

### Study design

We used a hybrid effectiveness-implementation cluster randomized trial design (the detailed research protocol is described elsewhere) [33]. GHs from six behavioral health agencies across eastern and central Massachusetts (Vinfen Corporation, Bay Cove Human Services, Advocates, North Suffolk Mental Health Association, Open Sky Community Services, and Riverside Community Care, Inc.) were randomized at a 1:1 ratio to “Tailored Best Practices” (TBP) or “General Best Practices” (GBP) in March 2021. A stratified block randomization scheme with block sizes of 4 was implemented [34]. Stratification factors included GH-level of race of GH staff and residents, a COVID-19 infection risk score and GH home division (ID/DD vs. SMI). GHs were stratified as Black vs. Others based on whether the home was among GHs with less or greater than the median proportion of residents and staff who were Black. GHs were stratified as high risk vs. low risk based on whether the home was among GHs with less or greater than the median proportion of residents and staff who were ‘immune’ based on prior infection and vaccination. All study staff other than those responsible for randomization were blinded. Ethical approval for this study was obtained from the Institutional Review Boards of Massachusetts General Brigham, Massachusetts Department of Developmental Services (DDS), and Massachusetts Department of Mental Health (DMH). Informed consent was obtained from all the participants and/or their legal guardians.

GHs with a focus on acute brain injury or substance abuse were excluded. The GHs were followed up at five regular 3-month intervals to assess effectiveness and implementation outcomes. Residents who lived in a GH and staff who worked in a GH at any time point during the study period were included in the sample, contributing to data at the corresponding intervals in which they lived or worked. A minority of residents and staff were known to have moved across different GHs and may have crossed between arms during study follow-up. A previous publication provides additional descriptions of the study sites and recruitment procedures [33].

### Study arms

GBP consisted of standard dissemination of best practices for preventing COVID-19 by the Centers for Disease Control and Prevention (CDC) and dissemination of required COVID-19 prevention practices by the Massachusetts Executive Office of Health and Human Services (MA HHS). Over the course of the study, training in GBP was provided by the behavioral health agencies consistent with state directives and regularly modified to reflect up-to-date recommendations consistent with CDC and MA HHS required standards. TBP included the GBP required prevention practices in addition to a four-component tailored implementation strategy delivered to both the group home residents and staff consisting of (1) trusted messengers (2), motivational interviewing; (3) interactive education; and (4) fidelity dashboard feedback (Supplementary Figure S1). The trusted messenger component included in-person and recorded peer testimonials on the benefits of vaccination, tapping into the potential effectiveness that peers with similar identities and experiences can bring as trusted sources of information on vaccination [35–37]. The motivational interviewing component was aimed at engaging residents with serious mental illness and group staff in considering and adopting preventive practices (i.e., screening, vaccination, face mask use, and hand hygiene). Motivational interviewing (MI) was selected based on evidence of effectiveness in promoting health behavior change in individuals with SMI [38, 39] and based on potential effectiveness in reducing vaccine hesitancy [40, 41]. Motivational interviewing was not offered to residents with ID/DD given the cognitive demands of this intervention. Interactive education was conducted using webinars and local discussions in GHs as a forum for disseminating and discussing the benefits of the preventive practices [42]. Finally, facilitated “House Plans” were held at 3-month intervals where GH Program Directors (PD) were provided with a numeric ‘fidelity dashboard’ outlining updates on their home’s estimated fidelity performance in comparison to their organization’s overall performance score. The dashboard contained information on the estimated proportion of staff and residents engaging in screening, vaccination, face mask use, and hand washing functioning, as a means to provide feedback to the agencies and GHs to optimize adherence, consistent with principles of audit and feedback [33, 43]. The TBP components were determined through a stakeholder co-design process including: (1) assembling a systematic review of evidence-based prevention strategies; (2) soliciting feedback from employees within participating provider organizations on the review and options; (3) conducting 36 key-informant interviews with staff and 24 key-informant interviews with residents on strategies to implement recommended best practices for COVID-19 prevention; (4) presentation of a validated simulation model determining the comparative effectiveness of different COVID-19 preventive practices; and (5) convening of a COVID-19 Quality Improvement Collaborative of a multi-stakeholder working group described above, to identify priorities for TBP. This process and implementation by four implementation coaches is described in detail in a previous publication [33]including delivery in three overlapping phases throughout the study period (Supplementary Figure S1).

## Measures

### Implementation outcomes

The primary implementation outcomes included full COVID-19 initial vaccination status among (1) residents and (2) staff who were unvaccinated during the baseline period (despite the promotion of COVID-19 vaccination by the participating organizations over the prior 4 months) and (3) fidelity to COVID-19 prevention practices (screening, vaccination, mask use, and handwashing) at GH-level (Supplementary Table S2). Subgroup implementation outcomes by race and ethnicity included full vaccination status among SMI and ID/DD residents, non-Hispanic White and non-White residents, and non-Hispanic White and non-White staff. Vaccination was added as a primary outcome to the study in March 2021. Individual-level dates of COVID-19 vaccinations were obtained from records maintained by GH organizations. A person was considered to be initially fully vaccinated when he or she received the full dosage of initial immunization(s) as recommended by the CDC during the study, either two initial doses of the Pfizer or Moderna vaccine or one dose of the Johnson & Johnson vaccine. Baseline vaccination rates were established from January 1, 2021, to March 31, 2021. Time to full vaccination was defined as the time from April 1, 2021 to the date of vaccination.

Fidelity was assessed via surveys designed for this study that consisted of anchored indicators of COVID-19 recommended preventive practices: masking, symptom screening, hand washing, vaccinations, and vaccination boosters (Supplementary Table S2) [33]. These surveys were completed by GH PDs over five regular 3-month intervals from August 2021 through October 2022 (baseline measures from May through July 2021). Since state and federal recommended COVID-19 prevention practices changed throughout the study period, the specific components of the overall fidelity score (ranging from 0 − 100%) differed over the study time points (Supplementary Table S3).

Secondary implementation outcomes, informed by the RE-AIM implementation framework [44–46], included acceptability (measured by the Acceptability of Intervention Measure), appropriateness (measured by the Intervention Appropriateness Measure), feasibility (measured by the Feasibility of Implementation Measure) [44–46], reach, and maintenance. Reach was defined as the proportion of the GHs receiving all four TBP components: Motivational Interviewing, Interactive Education, Trusted Messengers, and a facilitated House Plan for COVID-19 prevention practices. Maintenance was defined as the proportion of GHs who maintained a fidelity score > 80% at 15 months into the study (Supplementary Table S2).

### Effectiveness outcomes

The primary effectiveness outcomes included incident COVID-19 infections in GHs among (1) the overall sample of staff and residents combined (2), residents alone, and (3) staff alone. COVID-19 infections, tracked and reported by GH organizations, were directly tested in symptomatic residents or self-reported or directly tested by staff. COVID-19 infections were measured by incidence rates at each GH, defined as the number of incident cases divided by the total follow-up time (per 100 person-months) for each of the five 3-month periods over April 2021 through June 2022 (baseline measures from January through March 2021) and respective study sample. Resident follow-up time was defined as the number of months a resident spent living in a GH based on intake and discharge administrative data. Staff follow-up time was defined as the number of months a staff member had consistent shifts in a home with no gap in shifts greater than 30 days.

The secondary effectiveness outcomes included (1) COVID-19-related hospitalization among residents and (2) COVID-19-related deaths among residents (Supplementary Table S2). COVID-19-related hospitalizations and deaths were reported directly from participating GH provider organizations.

### Statistical analysis

The cumulative incidence of vaccinations over time were estimated using Kaplan-Meier curves by arm for the overall population, residents, and staff. Residents and staff were classified to intervention arms based on the GH they were associated with at the time of randomization. Only staff or residents present in the homes at baseline were included. Confidence intervals for cumulative incidence curves were calculated based on log transformation of the survival function and Greenwood’s formula. A Cox frailty model was fit to evaluate differences in the hazard of vaccination uptake between arms separately within resident and staff populations. These models included a main effect for intervention arm and additionally adjusted for stratification factors, GH agency, and GH-level log-normal frailties [47]. These analyses were repeated in additional subgroups in secondary analyses.

For the analysis of the fidelity scores and GH-level incidence of COVID-19 infections, analogous linear mixed models (LMMs) and Poisson generalized linear mixed models (GLMMs) were fit to evaluate differences in the mean trends of the scores and incidence rates over the five 3-month follow-up time periods. The models included main effects for intervention arm, linear and quadratic time trends, and intervention-by-time interaction terms. The models additionally adjusted for baseline fidelity score or infection rate, the stratification factors, agency, and a GH-level Gaussian random intercept term. GHs with missing baseline fidelity scores were excluded when fitting the model for fidelity. To assess whether mean trends differed between intervention arms based on these models, a joint test for whether any of the intervention main effect or intervention-by-time interaction effects differed from zero was conducted to. Bonferroni-adjusted p-values were reported for tests for the six primary outcomes to account for multiple testing. The adjusted marginal mean fidelity scores and incidence rates by time period and arm were calculated from the LMM and GLMM models to exhibit how mean scores and incidence varied over time by arm. Analysis of the secondary acceptability, appropriateness, feasibility, and reach outcomes followed the same approach as that of the fidelity score.

Treatment effect heterogeneity for the impact of the intervention on vaccine uptake by race/ethnicity and agency was assessed by refitting the Cox model to include corresponding main effects and interactions effects with intervention and testing for an interaction effect. Similarly, the GLMM for infection rates were refit to include corresponding main and interaction effects and jointly testing for whether any of the intervention-by-subgroup and intervention-by-time-by-subgroup interaction effects were non-zero. In additional analyses, GH-level fixed effects (e.g. number of bedrooms, intensity level, etc.) were added to the GLMM for infection rates to identify GH-level predictors of GH-level infection rates. P-values for analyses of secondary outcomes have not been corrected for multiplicity. A Poisson generalized linear model was used to assess hospitalization rates over the entire study period. The model included main effects for treatment group, fixed effects for randomization factors and GH agency, and a GH-level random intercept. Analyses were performed in Stata 16 (College Station, Texas) and R version 4.2.0 (Vienna, Austria).

For the continuous Best Practices Fidelity score, we had 80% power to detect minimum 0.21 standardized mean difference between intervention and control (Cohen’s d = 0.21). Given COVID-19 incidence rates as of May 2020 based on available COVID-19 prevalence data for GHs residents and staff, we had at least 80% power to detect 6–10% percentage point difference of COVID-19 infection between GBP and TBP. Further details on power calculations are described in a previous publication [33].

A Data and Safety Monitoring Board (DSMB) reviewed the unblinded data on the predetermined date of January 27, 2022. The DSMB was tasked with determining whether the study should proceed as planned, proceed with modifications, or terminate early if there was overwhelming effectiveness, futility, or safety issues. The DSMB determined that the study could proceed as planned.

## Results

### Sample characteristics

**Fig. 1:**
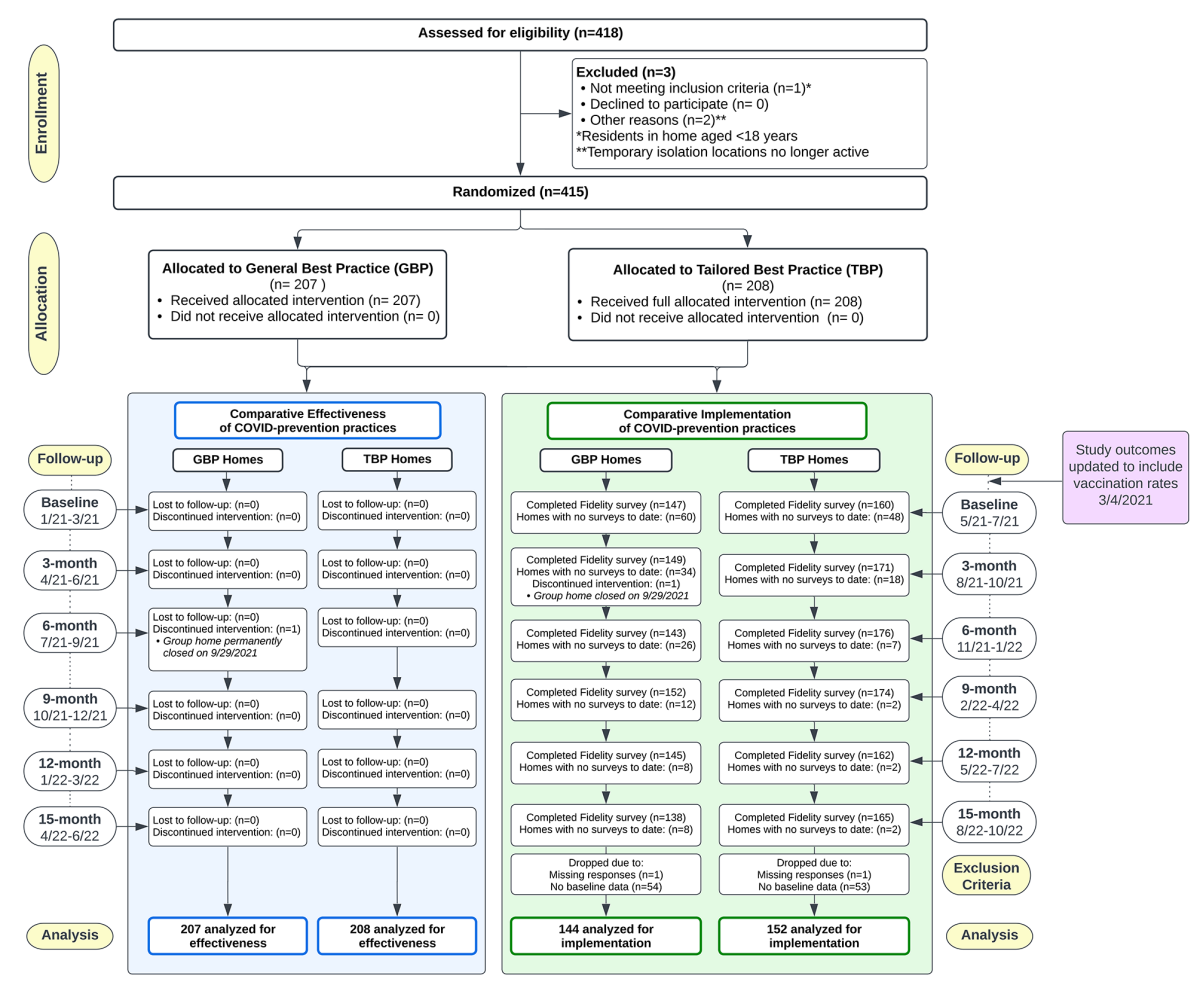
Participant CONSORT diagram

The study sample consisted of 415 GHs (208 randomized to TBP and 207 to GBP; Fig. 1) that included 3,836 residents (1,041 ID/DD and 2,795 SMI) and 5,538 staff. SMI residents had a mean age of 44 (SD = 15), were 64% male, and 59% non-Hispanic White. ID/DD residents had a mean age of 50 (SD = 17), were 57% male, and 70% non-Hispanic White. Staff had a mean age of 41 (SD = 13), were 59% female, and 71% non-Hispanic Black. The GH-level baseline characteristics were similar between study arms (Supplementary Table S4). Recruitment took place between November 2020 to December 2020, and the RCT was implemented from April 2021 to June 2022, coming to a close after 15 months as planned. Randomization was finalized April, 2021. There were no delays between randomization and initiation of the TBP.

### Implementation outcomes

#### COVID-19 vaccination rates

**Fig. 2:**
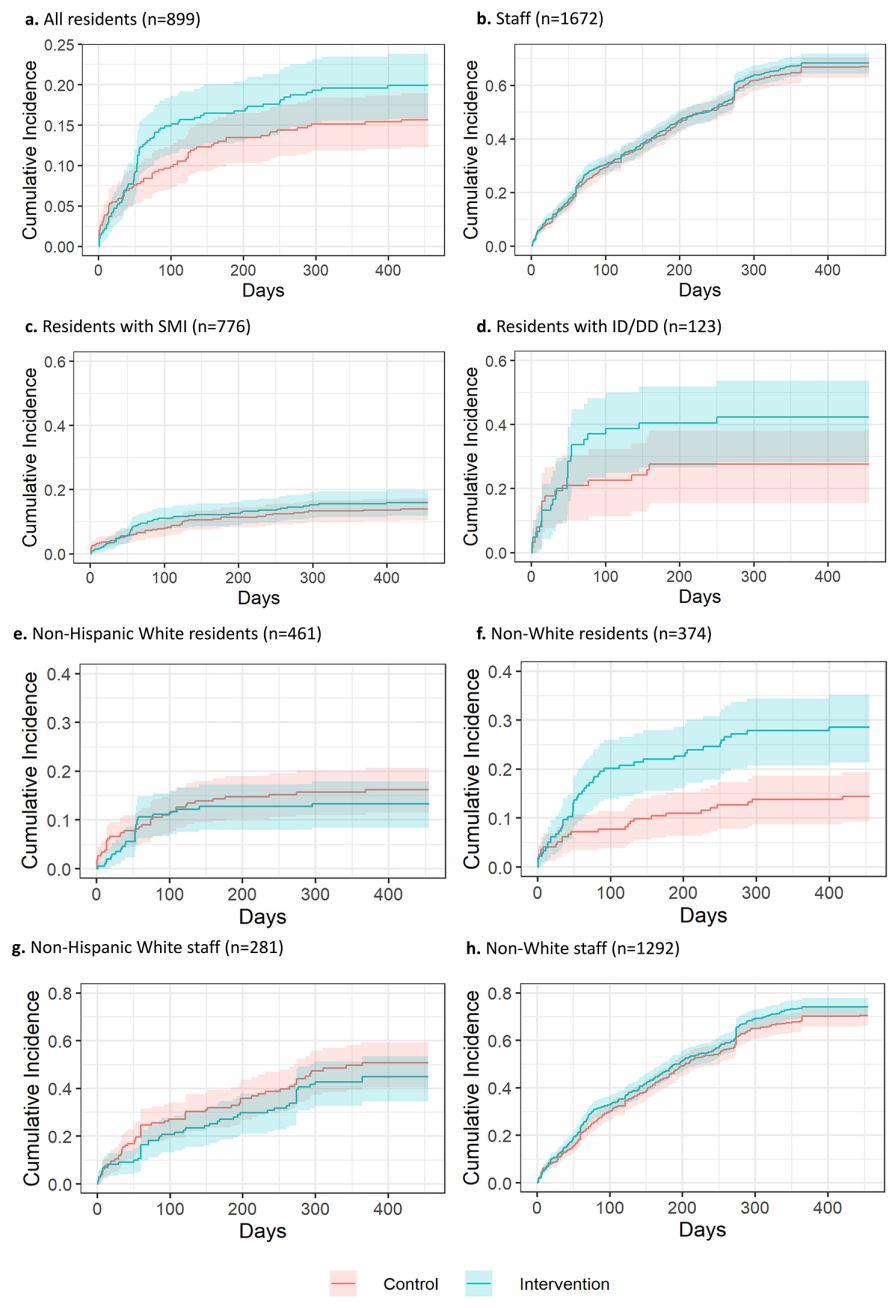
COVID-19 vaccination cumulative incidence curves. ^a^Figures 2a–h span 4/1/2021–6/30/2022. ^b^ID/DD = intellectual disabilities/developmental disabilities; SMI = serious mental illness. ^c^Non-White includes non-Hispanic (NH) Black, Hispanic, NH American Indian/Alaska Native, NH Asian, NH Two or More Races, NH Hawaiian/Other Pac Islander, Non-Hispanic Other (unspecified)

During the baseline period, 899 (32%) residents and 1,672 staff (46%) had not been fully vaccinated. Figure 2 shows the estimated cumulative incidence of becoming fully vaccinated among residents and staff. The rates of achieving full vaccination status over the follow-up did not significantly differ among residents who received TBP relative to GBP (HR = 1.21 relative to GBP, 95% CI = 0.79–1.84, Bonferroni-adjusted *p* > 0.99, Table 1; Fig. 2a, Supplementary Table S5A) and staff (HR = 0.99, 95% CI = 0.86–1.15, Bonferroni-adjusted *p* > 0.99, Table 1; Fig. 2b, Supplementary Table S5B).

**Table 1:** Estimates of intervention effect (TBP vs. GBP) on vaccination rates in overall populations and subgroups

Population	HR	*p*-value	95% CI
Primary Outcomes			
*All Residents*	1.21	0.38	(0.79, 1.84)
*All Staff*	0.99	0.91	(0.86, 1.15)
Subgroup Analyses			
*Residents with SMI*	1.07	0.78	(0.69, 1.65)
*Residents with ID/DD*	1.76	0.34	(0.55, 5.66)
*Non-Hispanic White Residents*	0.79	0.49	(0.40, 1.55)
*Non-White Residents*	2.25	0.001	(1.38, 3.68)
*Non-Hispanic White Staff*	0.70	0.07	(0.47, 1.03)
*Non-White Staff*	1.03	0.72	(0.88, 1.21)

*All models included a main effect for intervention arm and are adjusted for stratification factors used in randomization, GH agency, and GH-level log-normal frailties

*Acronyms*: TBP - Tailored Best Practices; GBP - General Best Practices; HR - Hazard Ratio; SMI - Serious Mental Illness; ID/DD - Intellectual Disabilities/Developmental Disabilities

In subgroup analyses, the cumulative incidence of achieving full vaccination status among non-White residents was estimated to be 28.6% in the TBP arm and 14.4% in the GBP arm at 15 months (Fig. 2e). The rate of achieving full vaccination status was higher for TBP in this subgroup (HR = 2.25, 95% CI = 1.38–3.68, *p* = 0.001, Table 1, Supplementary Table S6A). However, there were no differences in rates of vaccination among Non-Hispanic White residents (HR = 0.79, 95% CI = 0.40–1.55, *p* = 0.46, Table 1, Supplementary Table S6B, Fig. 2f). In treatment effect heterogeneity analyses, the effect of TBP on the rate of vaccination was higher among non-White than Non-Hispanic White residents (ratio of HR between non-White vs. Non-Hispanic-White: 2.28, *p* = 0.03, Table 1, Supplementary Table S6C). There were no significant effects among staff by race/ethnicity nor residents by GH type (Table 1; Fig. 2c-e, Supplementary Tables S6D, S7A–B).

#### COVID-19 best practices Fidelity

**Fig. 3:**
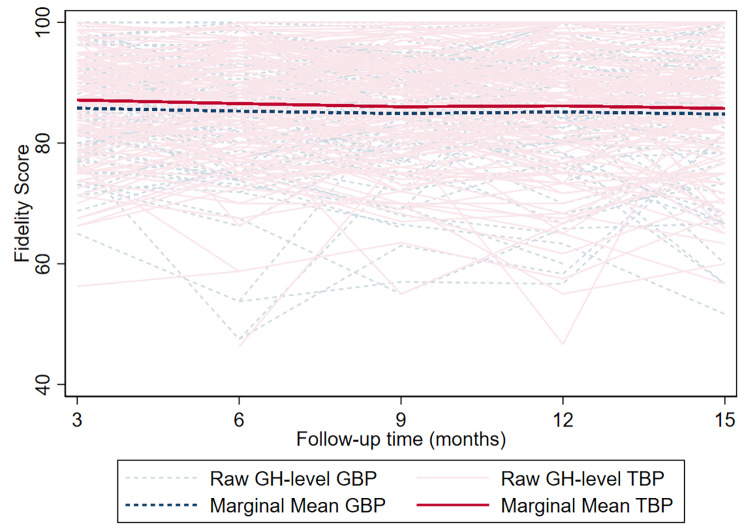
Group home-level marginal mean fidelity scores by study arm. ^a^Abbreviation: GBP, General Best Practice; TBP, Tailored Best Practice. ^b^Fidelity scores could range from 0 to 100. ^c^Month ranges represented on x-axis: 3, Aug ‘21 – Oct ‘21; 6, Nov ‘21 – Jan ‘22; 9, Feb ‘22 – Apr ‘22; 12, May ’22 – Jun’ 22; 15, Aug ‘22 – Oct-22. ^d^P-value is derived from a joint Wald test for the main and interaction effects of intervention testing for differences in fidelity scores between arms across time points

GBP homes (*n* = 54) and TBP homes (*n* = 53) with missing baseline fidelity scores were excluded when fitting the model. Mean trends in Fidelity score did not significantly differ, with scores estimated to be 0.92–1.37 points higher in the TBP arm (Bonferroni-adjusted *p* > 0.99; Fig. 3, Supplementary Tables S8A and S8B).

#### Adoption, reach, and maintenance

Marginal mean acceptability, appropriateness, and feasibility scores were higher among TBP homes relative to GBP homes over the follow-up. There were significant differences in the mean trends in each of these scores between arms based on the LMMs (test for intervention effect, *p* = 0.01, 0.005, 0.004 for acceptability, appropriateness, feasibility, respectively; Supplementary Materials, Sections S9, S10, S11). Reach was high: 90% (range 77–100% by agency) of GHs in the TBP arm received all four components of the intervention (Supplementary Tables S12A and S12B). Maintenance was also high. Of the 106 GHs receiving GBP and 126 GH receiving TBP who completed both the baseline and end-point survey, 79% and 77% maintained a fidelity score > 80% at the 15-month follow-up, respectively.

### Effectiveness outcomes

#### Laboratory-confirmed cases of COVID-19 infections

**Fig. 4:**
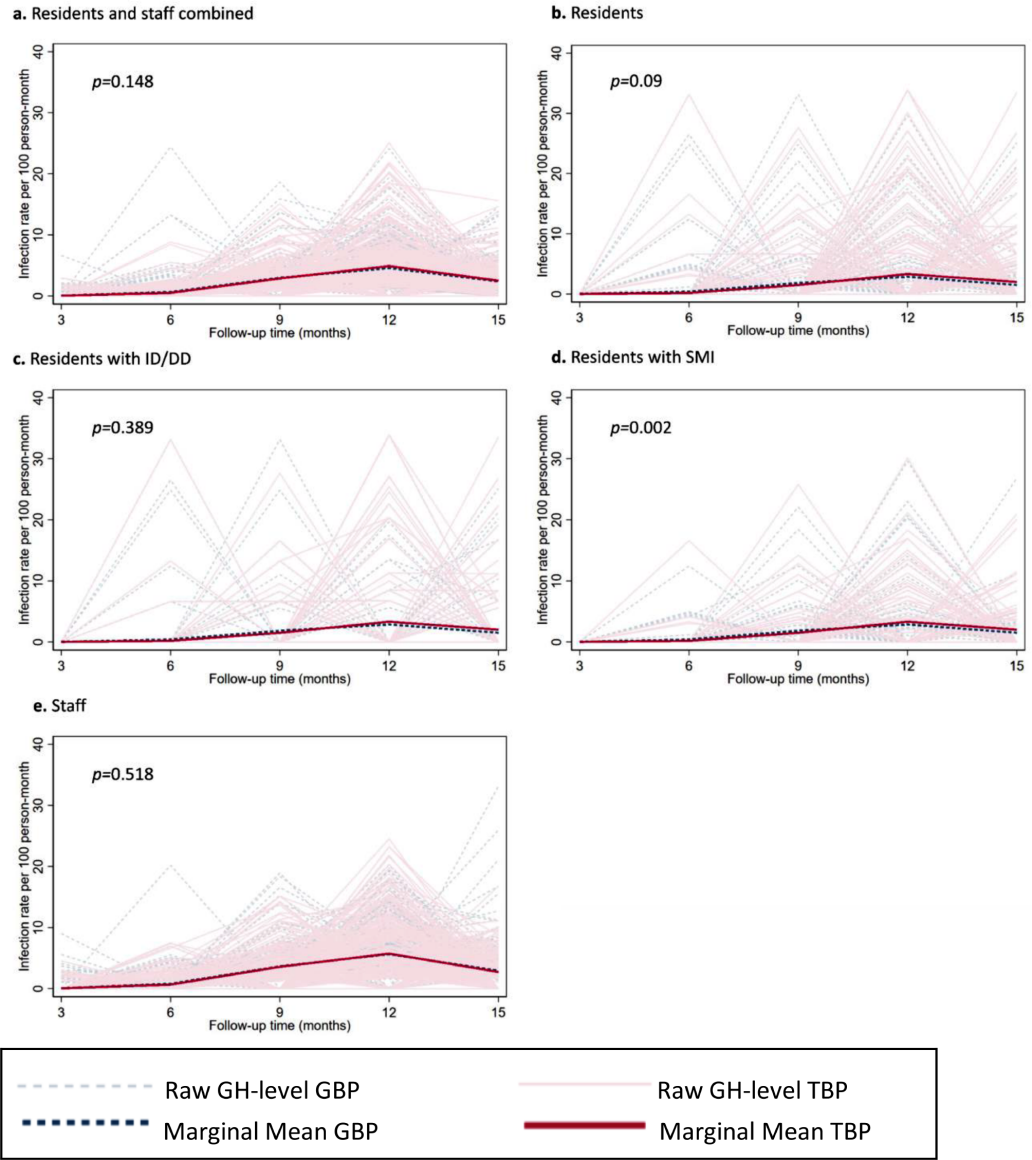
Marginal mean and raw GH-level COVID-19 incidence rates. Abbreviation: GH, Group home; GBP, General Best Practice; TBP, Tailored Best Practice. Note: Month ranges represented on x-axis: 3, Apr ‘21 – Jun ‘21; 6, Jul ‘21 – Sep ‘21; 9, Oct ‘21 – Dec ‘21; 12, Jan’22 – Mar’22; 15, Apr ‘22 – Jun-22

There were no significant differences in GH-level mean infection rate trends between arms among combined residents and staff population, resident population, or the staff population (Bonferroni-adjusted test for intervention effect *p* = 0.89, > 0.99, 0.09, respectively; Fig. 4a, Supplementary Tables S13A–F). In secondary analyses, trends in mean infection rate differed between TBP and GBP among SMI residents (test for intervention effect, *p* = 0.002, Fig. 4d, Supplementary Table S13G), such that TBP homes were associated with lower rates at earlier time periods (IRR: 0.32–0.90 over months 1–9 time points) and higher rates at later time points (IRR: 1.52–2.57 over months 10–15 time points, Supplementary Table S13H). The absolute differences between TBP and GBP were modest, at most around 7.5 additional cases of infections per 1,000 person months for TBP GHs at the month 13–15 time point. There were no significant differences in mean incidence trends among ID/DD residents (test for intervention effect, *p* = 0.39, Fig. 4c, Supplementary Tables S13I and S13J).

Overall, there were no significant heterogeneity in treatment effects by race for either residents and staff (Supplementary Tables S14A–D). Further subgroup analyses by race are described in Supplementary Materials, Section S15. Heterogeneity in mean home-level COVID-19 incidence rates for residents were observed across agencies, although the results did not have meaningful application (Supplementary Tables S16A–B, S17A–B). Further subgroup analyses by agency are described in Supplementary Tables S16C–H.

#### COVID-19-related hospitalization among residents

For all residents, rates of hospitalization were not estimated to be significantly different between TBP and GBP across the study period (IRR = 0.71, 95% CI = 0.33–1.53, *p* = 0.38) (Supplementary Table S18).

#### COVID-19-related deaths among residents

Both TBP and GBP had fewer than 10 COVID-related deaths. Per IRB protocol, cell sizes of 10 or less are not to be reported to maintain de-identification.

#### GH-level predictors of high COVID-19 infection rates

GH-level resident infection rates were inversely associated with prevalence of prior resident infection (IRR: 0.99 for GHs with a 1% higher baseline prevalence of prior infection, *p* ≤ 0.001, 95% CI: 0.98–0.99) and positively associated with prevalence of resident vaccination (IRR: 1.01 for homes with a 1% higher prevalence of vaccination, *p* = 0.02, 95% CI: 1.00-1.01). Further analyses are described in Supplementary Materials, Section S19.

## Discussion

This rapid randomized, pragmatic, hybrid effectiveness-implementation trial evaluated the effectiveness of tailored COVID-19 prevention strategies involving 3,836 residents with SMI and ID/DD and 5,538 staff of 415 GHs. TBP was associated with greater acceptability, appropriateness, feasibility, and reach as an implementation strategy than GBP. No differences were found in overall rates of vaccination, fidelity to COVID-19 prevention practices, or differences in the incidence of COVID-19 in a comparison of Tailored Best Practices (TBP) to General Best Practices (GBP). However, TBP compared to GBP was associated with greater vaccine acceptance among racially/ethnically diverse residents.

Acceptability, appropriateness, and feasibility was higher in TBP compared to GBP, which aligns with the intent of tailoring implementation strategies to improve health equity for health disparity populations and contexts [48, 49]. The selection of the four tailoring components was made by stakeholder workgroups charged with optimizing the implementation strategy to the setting and population. Of note, over 90% of the TBP GHs received all four components of the implementation strategy, providing additional support for acceptability, feasibility, and appropriateness. Our collaborative infrastructure of stakeholder workgroups, previously described [33], further contributes to existing evidence of the benefits of stakeholder engaged, co-designed implementation strategies in delivering evidence-based interventions to populations living with mental disabilities [50–52].

Our finding on the impact of TBP increasing vaccine acceptance among non-White mentally disabled adults has potential implications for addressing vaccine hesitancy among high-risk individuals. The subgroup of non-White unvaccinated residents in GHs had approximately double the rate of vaccination acceptance compared to non-Hispanic White residents at 15-month follow-up. Our finding of higher vaccination rates of non-White residents receiving TBP is noteworthy considering the intersectionality of “vaccine hesitancy” among individuals from ethnic and racial minorities and among adults with disabilities [27, 28, 30, 31, 53–55]. All participants had been previously offered COVID-19 vaccination by the six participating agencies 6 months before the initiation of our RCT. At baseline, 46% of staff and 32% of residents were not yet fully vaccinated for COVID-19 and were considered “vaccine-hesitant.” As non-White individuals and individuals with disabilities separately represent health disparity populations, this finding has relevance in the context of recent attention to the unique risk factors and vulnerabilities associated with intersectionality for the subgroup of non-White individuals with disabilities [53–55].

Our study is novel in evaluating the effectiveness of a tailored approach to COVID-19 prevention and vaccine acceptance in people with disabilities by conducting a randomized control trial comparing two different implementation strategies. The limited research literature on interventions aimed at increasing vaccination rates for adults with SMI consist of pre-post studies, including a pilot study of vaccine administration in a Clozapine clinic population in the context of close monitoring and routine laboratory tests that achieved 84% vaccination rates compared to the outpatient rates from 62 to 77% [29]. A pre-post study of a co-developed, culturally-tailored counseling educational intervention for underserved ethnic minority adults with psychiatric disorders found that among the 25.4% of clients (*n* = 16) in the sample who reported being unlikely to accept COVID-19 vaccination, following the intervention 17.5% (*n* = 13) reported remaining unlikely to be vaccinated [56]. Adults with ID/DD similarly have disproportionately high rates of vaccination hesitancy (including greater hesitancy for non-White individuals) and high rates of morbidity and mortality, especially early in the pandemic, compared to the general population [27, 57]. A large study of health care workers reported the greatest vaccine hesitancy for individuals who are Black and Hispanic/Latino, underscoring the need to develop effective strategies for implementing vaccines in frontline vaccine hesitant health care workers [31].

Higher and more rapid attainment of COVID-19 vaccination among non-White residents for our TBP implementation strategy suggests that tailoring may benefit among the most vaccine hesitant subgroups who represent minoritized populations and who have mental disabilities. In contrast, as both TBP and GBP GHs demonstrated an overall similar increase in vaccination, tailoring may not confer additional benefit overall for the general population of residents and staff in these settings.

That greater vaccine acceptance was found for non-White residents suggests that the selected implementation strategy may have a unique cultural and/or social resonance. The use of “trusted messengers” consisting of peer advocates and recorded peer testimonials has been recommended as a potentially valuable strategy for improving COVID-19 vaccination rates among racially diverse subgroups [35–37]. However, as all four components of our multi-component implementation strategy (motivational interviewing, interactive education, trusted messengers, and fidelity dashboard feedback) were distributed at overlapping times, we are unable to distinguish which components might have been most influential. Similarly, we are not able to evaluate the unique contribution of fidelity dashboard feedback. Future research may benefit from testing the effectiveness of individual components (e.g., trusted messengers or fidelity dashboard feedback) compared to other strategies. Greater vaccine acceptance was seen among residents but not staff, suggesting that this implementation strategy may have had a unique resonance among ethnically diverse individuals with disabilities, potentially contributing to addressing the unique challenges of addressing intersectionality in health disparities.

Despite successfully engaging six behavioral health agencies in a co-designed implementation process for evidence-based COVID-19 prevention practices, we did not find differences between TBP and GBP with respect to overall fidelity to recommended COVID-19 prevention practices. During the course of the study, a variety of different practices believed to prevent COVID-19 infections were promoted at the state and federal level including symptom screening, use of face masks, hand washing, and COVID-19 vaccination. As scientific understandings sharpened and governmental policy recommendations evolved, the requirements for these preventative practices changed—and even varied—between GH agencies. Accounting for these changes, the overall fidelity to these preventative practices was high, though not different between the GHs receiving TBP and GBP. This lack of statistical significance with respect to fidelity between these arms might be explained by a combination of several factors. First, by the time our intervention was delivered in April 2021, the COVID-19 pandemic had already been underway for more than one year. As such, most of these preventative practices were already being implemented in the GH settings, and a substantial proportion of residents and staff had a prior COVID-19 infection conferring immunity, limiting the potential for significant improvement, as evidenced from the high-fidelity scores at baseline. Second, the TBP arm included best practices mandated by state officials in the Massachusetts Department of Health and Human Services. These mandates, alone, might have been sufficiently influential in the absence of tailoring, making incremental improvement in fidelity difficult to detect. In addition, one of the agencies required vaccination of staff, potentially contributing to the lack of a difference found between TBP and GBP for vaccinations for staff. Third, the fidelity to these preventative practices were self-scored by the directors of the GHs. A positive response bias could have equally impacted both arms. Independent measurement of the preventative practices was not possible due to state-mandated isolation and social distancing precautions that were in place during much of the study.

Of note, we did not identify any meaningful differences in COVID-19 infection rates between arms for our main populations (staff, residents) or subgroups (e.g., SMI and ID/DD; non-White and non-Hispanic White). However, of interest, there was a Group-by-Time finding for the SMI population such that TBP v. GBP was associated with lower incidence of COVID-19 over the first 9-month time points, followed by higher rates at later time points (months 10–15). This finding may reflect the initial benefits of relative isolation for this subgroup earlier in the pandemic with the combination of preventive strategies (e.g., mask use, social distancing, screening, and vaccination) [58]. Then, when uninfected individuals increasingly ventured out into the community, they had exposures to COVID-19. This reflected the emerging understanding that vaccination was mostly associated with *decreasing the severity* of COVID-19 symptoms, but not *preventing infection* [59]. Our results were consistent with this general finding as resident hospitalization and mortality from COVID-19 was minimal to negligible in our sample across both arms, also reflecting the evolution of less virulent COVID-19 variants. By the time our intervention had started, the majority of staff and residents had also already been fully vaccinated, and outpatient treatment options for COVID-19 were well underway. In addition, at least 19% of residents and 17% of staff present at baseline had a prior episode of COVID-19 infection, potentially conferring natural immunity and further limiting the impact of additional vaccination.

As a rapid, pragmatic, randomized prevention trial occurring during an evolving pandemic, our study has numerous limitations that should be considered in interpreting the results. As described above, we introduced a tailored implementation strategy for COVID-19 prevention in the midst of the COVID-19 pandemic after initial efforts to prevent COVID-19 had already been implemented by state authorities (GBP) and after a substantial subgroup of residents and staff had already experienced COVID-19 infection. In addition, the RCT occurred in the context of rapidly evolving and changing recommended practices, prevention policies, and base-rates of infection. To accommodate these dynamic and rapidly changing contexts, we incorporated adjustments for major changes in external factors such as treatment standards, policies, and changing base rates and nature of the target (e.g., COVID-19 rates and variants). A possibility remains that these adjustments masked true effects.

The design of this study evaluated outcomes using a multi-component tailored implementation strategy compared to general best practices mandated by the Massachusetts Department of Health and Human Services. As we observed improvements over time in both study arms, it is possible that the policies and practices in the GBP arm of our study represent a highly effective approach and may not necessarily be representative of other states that had significantly lower base rates of immunizations or higher rates of COVID-related mortality. Our study also did not have the capacity to measure improvement in symptom severity that may reflect vaccination, beyond the extreme measures of hospitalization and death, which were already at low rates in Massachusetts at the start of our study trial. Finally, a complicating factor in measuring staff outcomes was a high rate of staff vacancies and turnover in the workforce underscoring the need for research that can address and prevent burnout and turnover in this high-risk, ethnically diverse, and socioeconomically challenged workforce [60].

Despite these limitations, there are many strengths including the scope, focus, innovation, and rapid responsiveness of the study to a public health emergency. We successfully engaged six different human service organizations across the state of Massachusetts in a participatory process of rapid co-design and collaborative execution of a COVID-19 prevention randomized control study of 415 GHs for adults with SMI and ID/DD in that included 3,836 residents and 5,538 staff. Our study was distinguished by including residents with different conditions (SMI and ID/DD) and the front-line provider workforce with significant ethnic and racial diversity allowing for the subgroup analysis revealing a differential benefit of tailored implementation in achieving greater COVID-19 vaccine acceptance among non-White mentally disabled adults. The GHs represented different geographic settings including urban, suburban, and more rural settings. The selection of the primary COVID-19 prevention practices was informed by an innovative use of simulation modeling coupled with a community and stakeholder engaged process. Finally, the design, recruitment, and execution of a cluster randomized implementation study was completed in a highly accelerated, adaptive process over just 2 years, reaching 90% of the GHs with a multi-component implementation strategy in the midst of a global pandemic, illustrating the feasibility of rapid, responsive implementation science responding to a public health emergency.

Our study underscores the need for additional future research to identify effective interventions and implementation strategies for the prevention of infectious disease and future pandemics, especially for racially and ethnically diverse disabled adults with SMI and ID/DD who are overrepresented among vaccine-hesitant individuals and have a reduced life expectancy. Specifically, our findings underscore the challenges and opportunities for addressing intersectionality in mitigating health disparities experienced by minoritized, disabled, low-income individuals at high risk of inadequate preventive care and poor health outcomes.

### Electronic supplementary material

Below is the link to the electronic supplementary material.

Supplementary Material 1

## Data Availability

All data collected as part of this study will be stored in a data repository at the Inter-university Consortium for Political and Social Research at the University of Michigan. The data that supported the findings from this study are available from the Inter-university Consortium for Political and Social Research at the University of Michigan upon reasonable request. Ethical approval may be obtained via formal application to the ICPSR for a specific project. The ICPSR website (https://www.icpsr.umich.edu/web/pages/ICPSR/access/restricted/) has full instructions.

## Abbreviations

SMI: Serious Mental Illness
ID/DD: Intellectual and developmental disabilities
GH: Group Home
TBP: Tailored Best Practices
GBP: General Best Practices
