# Supplementary material for: Tailored vs. General COVID-19 prevention for adults with mental disabilities residing in group homes: a randomized controlled effectiveness-implementation trial
Source: BMC Public Health. 2024 Jun 26;24:1705. doi: 10.1186/s12889-024-18835-w (PMC11201789; doi:10.1186/s12889-024-18835-w)
Supplement: Supplementary file 1 — Supplementary Material 1 [file 12889_2024_18835_MOESM1_ESM.pdf]

Tailored vs. General COVID-19 Prevention for Adults with Mental Disabilities Residing in Group Homes: A Randomized Controlled Effectiveness-Implementation Trial

SUPPLEMENTAL MATERIALS

Stephen Bartels[a, b,*],MD, MS; Julie H. Levison[a,b] , MD, MPhil, MPH; Hao D. Trieu[a], MPH; Anna Wilson[a], MPH; David Krane[a]; David Cheng, PhD[c]; Haiyi Xie[d], PhD; Karen Donelan[a], ScD, EdM; Bruce Bird[h], PhD; Kim Shellenberger[h], MBA; Elizabeth Cella[h], MS; Nicolas M. Oreskovic[b,f,l], MD, MPH; Kelly Irwin[g], MD, MPH; Kelly Aschbrenner[e], PhD; Ahmed Fathi[h], MPH; Stefanie Gamse[h] MPH; Sibyl Holland[h], MA; Jessica Wolfe[h], MA, MPH; Cindy Chau[a]; Adeola Adejinmi, MSN, BSN, RN[i]; Jasmine Langlois, MSN[j]; Jean-Louise Reichman, MA[k]; Lisa I. Iezzoni[a], MD, MSc; and Brian G. Skotko[f,l], MD, MPP

[a]Harvard Medical School, Massachusetts General Hospital, Mongan Institute, 100 Cambridge St, Suite 1600, Boston, MA, USA, 02114
[b]Harvard Medical School, Massachusetts General Hospital, Department of Medicine, 55 Fruit St, Gray 7-730, Boston, MA, USA, 02114
[c]Harvard Medical School, Massachusetts General Hospital, Department of Biostatistics, 50 Staniford Street, Suite 560, Boston, MA, USA, 02114
[d]Geisel School of Medicine at Dartmouth, Department of Biomedical Data Science, Williamson Translational Research Building, Third Floor, HB 7261, 1 Medical Center Drive, Lebanon, NH, USA, 03756
[e]Geisel School of Medicine at Dartmouth, Dartmouth-Hitchcock Medical Center, Department of Psychiatry, One Medical Center Drive, Lebanon, NH, USA, 03756
[f]Down Syndrome Program, Division of Medical Genetics and Metabolism, Department of Pediatrics, Massacuhusetts General Hospital, 125 Nashua Street, Suite 821, Boston, MA 02114, USA
[g]Harvard Medical School, Massachusetts General Hospital, Department of Psychiatry, 55 Fruit Street, Boston, MA, USA, 02114
[h]Vinfen Corporation, 950 Cambridge Street, Cambridge, MA, USA, 02141
[i]Bay Cove Human Services, 66 Canal Street, Boston, MA, 02114, USA
[j]Advocates Inc. 1881 Worcester Rd. Framingham, MA 01701, USA
[k]North Suffolk Mental Health Association, 301 Broadway, Chelsea, MA, 02150, USA
[l]Harvard Medical School, Massachusetts General Hospital, Department of Pediatrics, 55 Fruit Street, Boston, MA, USA, 02214

*Corresponding Author:
Stephen Bartels, MD, MS
Mongan Institute
Massachusetts General Hospital
Harvard Medical School
100 Cambridge St, Suite 1600
Boston, MA 02114
United States
Phone: +1 617-726-5213
Email: sjbartels@mgh.harvard.edu

Supplementary Figure S1. Tailored Best Practices component delivery timeline.......................... 6
Supplementary Table S2. Summary of primary and secondary effectiveness and implementation outcomes 7
Supplementary Table S3. Relevant Fidelity measures by wave, based on current COVID-19 policies 8
Supplementary Table S4. Home-level baseline characteristics.. 9
Supplementary Table S5. Primary Implementation Outcome: Association of study arm with home-level vaccination uptake 11
Supplementary Table S5A. Residents (IDD and SMI combined): Association of study arm with home-level time vaccination uptake between 4/1/2021-7/31/2022 (n=899) 11
Supplementary Table S5B. Staff: Association of study arm with home-level vaccination uptake between between 1/2021–7/2022 (n=1672) 12
Supplementary Table S6. Association of study arm with home-level vaccination uptake by race 13
Supplementary Table S6A. Non-White Residents: Association of study arm with vaccination uptake (n=374, subgroup analysis) 13
Supplementary Table S6B. Non-Hispanic White Residents: Association of study arm with vaccination uptake (n=461, subgroup analysis) 14
Supplementary Table S6C. Residents: Treatment effect heterogeneity on vaccine uptake by race 4/1/2021–6/30/2022 (n= 899) 15
Supplementary Table S6D. Staff: Treatment effect heterogeneity on vaccine uptake by race between 4/1/2021-6/30/2022 (n = 938) 16
Supplementary Table S7. Association of study arm with home-level vaccination uptake among residents by group home type 17
Supplementary Table S7A. Residents with ID/DD: Association of study arm with home-level time to vaccination between 1/2021–7/2022 (n=123) 17
Supplementary Table S7B. Residents with SMI: Association of study arm with home-level time to vaccination between 1/2021–7/2022 (n=776) 17
Supplementary Table S8. Primary Implementation Outcome: COVID-19 Best Practices Fidelity 18
Supplementary Table S8A. Association of GBP and TBP practices with Fidelity scores (n=296) 18
Supplementary Table S8B. Group-home-specific fidelity scores by time point 19
Supplementary Table S9. Acceptability... 20
Supplementary Table S9A. Association of GBP and TBP with group home-level Acceptability scores (n=278) 20
Supplementary Table S9B. Group-home-specific Acceptability scores by time point 21
Supplementary Figure 9C. Marginal mean Acceptability scores by study arm (zoomed scale) (n=278) 21

Supplementary Table S10. Appropriateness 22
Supplementary Table S10A. Association of GBP and TBP with group home-level Appropriateness (n=279) 22
Supplementary Table S10B. Group-home-specific staff Appropriateness scores by time point 23
Supplementary Figure S10C. Marginal mean Appropriateness scores by study arm (zoomed scale) (n=279) 23
Supplementary Table S11. Feasibility 25
Supplementary Table S11A. Association of GBP and TBP with group home-level Feasibility (n=278) 25
Supplementary Table S11B. Group-home-specific staff Feasibility scores by time point 26
Supplementary Figure 11C. Marginal mean Feasibility scores by study arm (zoomed scale) (n=278) 26
Supplementary Table S12. Reach 27
Supplementary Table S12A. Tailored Best Practices Implementation Reach 27
Supplementary Table S12B. Proportion of Group homes with complete and partial intervention completion by agency 27
Supplementary Table S13. Primary Effectiveness Outcome: Associations of GBP and TBP with group home-level COVID-19 incidence by time point 28
Supplementary Table S13A. Residents and staff combined: Associations of GBP and TBP with group home-level COVID-19 incidence (n=415) 28
Supplementary Table S13B. Residents and staff combined: Associations of GBP and TBP with group home-level COVID-19 incidence by time point 29
Supplementary Table S13C. Staff: Associations of GBP and TBP with group home-level COVID-19 incidence (n=415) 30
Supplementary Table S13D. Staff: Associations of GBP and TBP with group home-level COVID-19 incidence by time point 31
Supplementary Table S13E. Resident (ID/DD and SMI combined): Associations of GBP and TBP with group home-level COVID-19 incidence (n=415) 32
Supplementary Table S13F. Resident (ID/DD and SMI combined): Associations of GBP and TBP with group home-level COVID-19 incidence by time point 33
Supplementary Table S13G. Residents with SMI: Associations of GBP and TBP with group home-level COVID-19 incidence (n=209) 34
Supplementary Table S13H. Residents with SMI: Associations of GBP and TBP with group home-level COVID-19 incidence by time point 35
Supplementary Table S13I. Residents with ID/DD: Associations of GBP and TBP with group home-level COVID-19 incidence (n=206) 36

Supplementary Table S13J. Residents with ID/DD: Associations of GBP and TBP with group home-level COVID-19 incidence by time point 37
Supplementary Table S14. Group home-level COVID-19 incidence rate ratios by time point among residents and staff, by race/ethnicity 38
Supplementary Table S14A. Treatment effect heterogeneity on COVID-19 incidence by race, residents 38
Supplementary Table S14B. Group home-level COVID-19 incidence rate ratios by time point among residents, by race/ethnicity 40
Supplementary Table S14C. Treatment effect heterogeneity on COVID-19 incidence by race, staff 41
Supplementary Table S14D. Group home-level COVID-19 incidence rate ratios by time point among staff, by race/ethnicity 43
Supplementary Table S15. Group home-level incidence rate ratios by time point among residents and staff of SMI homes, by race/ethnicity 44
Supplementary Table S15A. Treatment effect heterogeneity on COVID-19 incidence by race, SMI residents 44
Supplementary Table S15B. Associations of GBP and TBP with group home-level COVID-19 incidence by time point among SMI residents, by race/ethnicity 45
Supplementary Table S16. Treatment effect heterogeneity on COVID-19 incidence by agency and time point among residents and staff........ 47
Supplementary Table S16A. Resident: Treatment effect heterogeneity on COVID-19 incidence by agency 47
Supplementary Table S16B. Association of GBP and TBP with group home-level COVID-19 incidence by time point among residents, by agency 49
Supplementary Table S16C. Staff: Treatment effect heterogeneity on COVID-19 incidence by agency 51
Supplementary Table S16D. Association of GBP and TBP with group home-level COVID-19 incidence by time point among staff, by agency 53
Supplementary Table S16E. Residents with IDD: Treatment effect heterogeneity on COVID-19 incidence by agency 55
Supplementary Table S16F. Association of GBP and TBP with group home-level COVID-19 incidence by time point among ID/DD residents, by agency 57
Supplementary Table S16G. Residents with SMI: Treatment effect heterogeneity on COVID-19 incidence by agency 59
Supplementary Table S16H. Association of GBP and TBP with group home-level COVID-19 incidence by time point among SMI residents, by agency 61
Supplementary Table S17. Sensitivity Analysis excluding Agency 5........ 63

Supplementary Table S17A. Resident (ID/DD and SMI combined) excluding Agency 5: Associations of GBP and TBP with group home-level COVID-19 incidence (n=385) 63

Supplementary Table S17B. Resident (ID/DD and SMI combined) excluding Agency 5: Associations of GBP and TBP with group home-level COVID-19 incidence by time point (n=385) 64

Supplementary Table S18. Association of GBP and TBP practices with home-level resident hospitalization rates between 1/2021-7/2022 (n=415) ... 65

Supplementary Table S19. Association of home-level characteristics with home-level COVID-19 infection incident rates ... 66

Supplementary Table S19A. Resident: Association of home-level characteristics with home-level COVID-19 infection incident rates (n=415) 66

Supplementary Table S19B. Residents with IDD: Association of home-level characteristics with home-level COVID-19 infection incident rates (n=206) 69

Supplementary Table S19C. Residents with SMI: Association of home-level characteristics with home-level COVID-19 infection incident rates (n=209) 71

Supplementary Table S19D. Staff: Association of home-level characteristics with home-level COVID-19 infection incident rates (n=415) 73

Supplementary Figure S1. Tailored Best Practices component delivery timeline

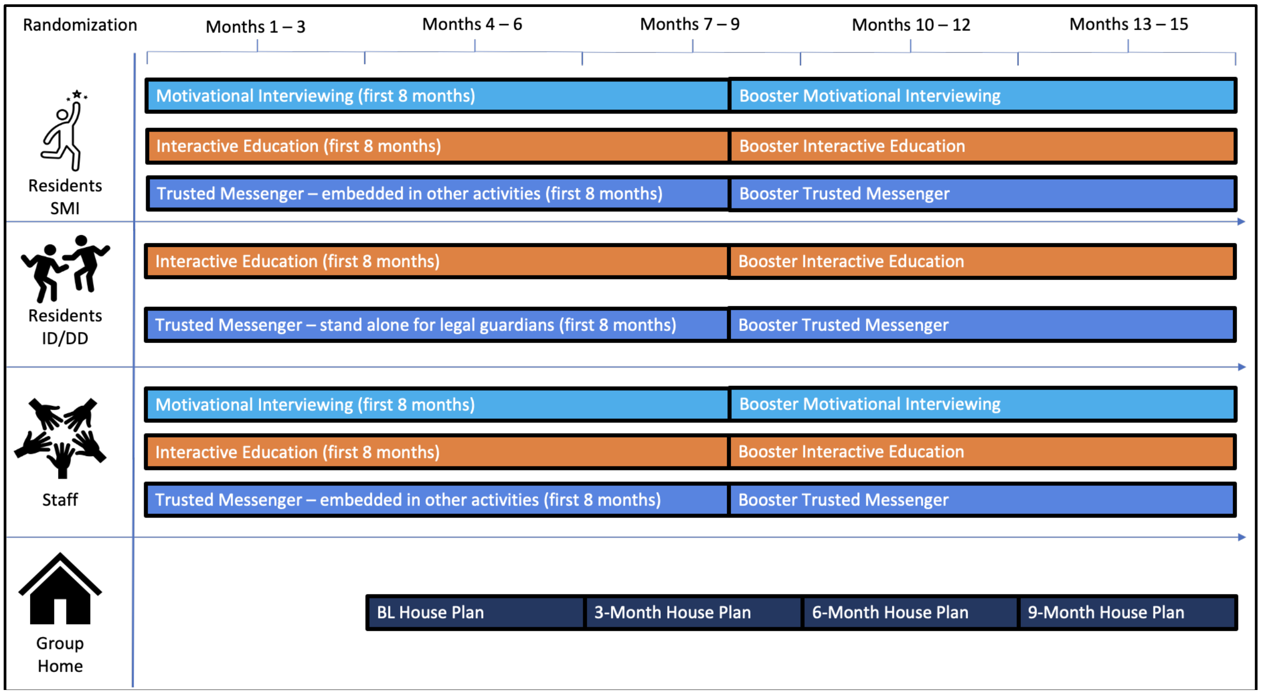

Supplementary Table S2. Summary of primary and secondary effectiveness and implementation outcomes

Implementation Outcomes		**Description**	**Source**	**Frequency**
Primary implementation outcomes:	COVID-19 Vaccination[a]	Receipt of COVID-19 vaccination	Secondary data routinely collected by the 6 participating provider organizations	Every 3 months beginning from initial introduction of the COVID-19 vaccine
	COVID Best Practices Fidelity	A score calculated based on GH engagement in up to 9 COVID-19 prevention activities (described below)	Surveys of GH Program Directors	Every 3 months beginning at baseline
Secondary implementation outcomes:	Adoption	Three separate scores calculated for Acceptability, Appropriateness, and Feasibility measures informed by the RE-AIM implementation framework[107]	Surveys of GH Program Directors	Every 3 months beginning at baseline
	Reach	The percentage of GHs with at least 80% COVID Best Practices Fidelity	Surveys of GH Program Directors	Every 3 months beginning at baseline
	Maintenance	The percentage of GHs maintaining at least 80% COVID Best Practices Fidelity over the 15-month study period	Surveys of GH Program Directors	Once at the end of the 15-month trial

Effectiveness Outcomes		**Description**	**Source**	**Frequency**
Primary effectiveness outcome:	COVID-19 Infection	New incidence of COVID-19 from laboratory testing of residents and staff	Secondary data routinely collected by the 6 participating provider organizations	Every 3 months beginning at baseline
Secondary effectiveness outcomes:	Hospitalization	New hospitalizations of GH residents due to COVID-19	Secondary data routinely collected by the 6 participating provider organizations	Every 3 months beginning at baseline
	Mortality	New deaths of GH residents due to COVID-19	Secondary data routinely collected by the 6 participating provider organizations	Every 3 months beginning at baseline

[a]Vaccination outcomes were added in March 2021 once they became publicly available.

Supplementary Table S3. Relevant Fidelity measures by wave, based on current COVID-19 policies

	Vaccinations		Boosters		Screening		Hand Hygiene		Masking	
Time	**Residents**	**Staff**	**Residents**	**Staff**	**Residents**	**Staff**	**Residents**	**Staff**	**Residents**	**Staff**
Baseline	☑	☑			☑	☑	☑	☑	☑	☑
3-month follow-up	☑	☑			☑	☑	☑	☑		☑
6-month follow-up	☑	☑			☑	☑	☑	☑		☑
9-month follow-up	☑	☑	☑	☑	☑	☑	☑	☑		☑
12-month follow-up	☑	☑	☑	☑			☑	☑		☑
15-month follow-up	☑	☑	☑	☑			☑	☑		☑

Supplementary Table S4. Home-level baseline characteristics

		Overall Sample		Residents		Staff	
Characteristics		**GBP (n=207)**	**TBP (n=208)**	**GBP**	**TBP**	**GBP**	**TBP**
**Age *mean (sd)***		44.3 (5.4)	43.7 (5.0)	48.8 (11.6)	48.1 (11.0)	42.2 (4.7)	41.9 (4.9)
**Sex *mean % (sd)***	Female	49.4 (21.7)	49.0 (23.2)	36.7 (34.0)	38.4 (35.3)	56.7 (21.1)	55.1 (22.4)
	Male	49.4 (21.7)	50.1 (23.2)	60.4 (34.1)	59.6 (34.9)	43.0 (21.0)	44.6 (22.5)
	Other/missing[b]	1.2 (2.8)	0.9 (2.6)	2.9 (8.5)	2.1 (6.5)	0.3 (1.6)	0.3 (1.3)
**Race & ethnicity *mean % (sd)***	NH White	34.5 (22.2)	31.5 (21.0)	64.3 (30.0)	59.9 (30.9)	22.0 (25.1)	19.1 (22.7)
	NH Black or African American	52.8 (23.1)	56.3 (22.4)	17.9 (25.0)	21.3 (26.9)	68.1 (27.4)	72.3 (25.0)
	Hispanic[c]	4.0 (5.8)	4.6 (7.2)	6.7 (12.2)	7.1 (12.3)	2.8 (5.8)	3.6 (7.6)
	NH Other[d]	4.1 (7.4)	4.2 (7.5)	4.8 (10.7)	6.5 (12.4)	3.7 (8.2)	3.1 (7.1)
	Missing	4.6 (6.5)	3.3 (5.1)	6.3 (12.2)	5.3 (9.3)	3.4 (7.8)	1.9 (4.7)
**Division *n (%)***	IDD	103 (49.8)	103 (49.5)				
	SMI	104 (50.2)	105 (50.5)				
**Agency *n (%)*[e]**	Agency 1						
	Agency 2						

Agency 3				
Agency 4				
Agency 5				
Agency 6				

Abbreviations: GBP, General Best Practice; TBP, Tailored Best Practice; SD, standard deviation; NH, Non-Hispanic; IDD, Intellectual and Developmental Disability; SMI, Serious Mental Illness

[a] Joint Wald tests were used to compare characteristics across arms

[b] "Other" sex category includes transgender, non-binary

[c] Ethnicity was not recorded separately from race by all agencies and thus cannot be reported as a separate category.

[d] "NH Other" includes non-Hispanic Asian, American Indian or Alaska Native, Hawaiian or Other Pacific Islander, Two or More Races, and unspecified Other

[e] Here and throughout: The GH number of individual agencies are suppressed to maintain their anonymity. Total number of GHs were in increasing numbers: 30, 34, 59, 62, 80, and 150.

Supplementary Table S5. Primary Implementation Outcome: Association of study arm with home-level vaccination uptake

Supplementary Table S5A. Residents (IDD and SMI combined): Association of study arm with home-level time vaccination uptake between 4/1/2021-7/31/2022 (n=899)

Fixed effects	HR	SE	*p*-value	95% CI
Intervention arm	1.21	0.22	>.99[a]	(0.79, 1.84)
Stratum 2	2.61	0.61	0.12	(0.79, 8.66)
Stratum 3	1.50	0.64	0.53	(0.43, 5.29)
Stratum 4	0.93	0.72	0.92	(0.23, 3.85)
Stratum 5	0.35	0.62	0.09	(0.10, 1.18)
Stratum 6	0.52	0.57	0.25	(0.17, 1.60)
Stratum 7	0.53	0.60	0.29	(0.16, 1.71)
Stratum 8	0.47	0.58	0.19	(0.15, 1.46)
Agency 1	1.85	0.38	0.10	(0.88, 3.89)
Agency 2	3.90	0.31	< 0.001	(2.12, 7.18)
Agency 3	2.52	0.43	0.03	(1.09, 5.86)
Agency 4	3.76	0.37	< 0.001	(1.82, 7.77)
Agency 5	3.34	0.53	0.02	(1.19, 9.41)

[a] Pvalue was adjusted for multiple comparisons using the Bonferroni correction.

Supplementary Table S5B. Staff: Association of study arm with home-level vaccination uptake between between 1/2021–7/2022 (n=1672)

Fixed effects	HR	SE	*p*-val	95% CI
Intervention arm	0.99	0.07	>.99[a]	(0.86, 1.15)
Stratum 2	1.22	0.13	0.13	(0.94, 1.60)
Stratum 3	1.1	0.16	0.56	(0.80, 1.50)
Stratum 4	0.78	0.16	0.11	(0.58, 1.06)
Stratum 5	1.28	0.16	0.12	(0.94, 1.74)
Stratum 6	1.18	0.14	0.24	(0.89, 1.57)
Stratum 7	0.68	0.19	0.04	(0.46, 0.99)
Stratum 8	1.34	0.15	0.05	(1.00, 1.79)
Agency 1	1.37	0.11	<0.01	(1.10, 1.70)
Agency 2	0.57	0.11	<.001	(0.45, 0.71)
Agency 3	0.94	0.12	0.57	(0.74, 1.18)
Agency 4	0.37	0.17	<.001	(0.26, 0.52)
Agency 5	1.18	0.14	0.23	(0.90, 1.56)

[a] Pvalue was adjusted for multiple comparisons using the Bonferroni correction.

Supplementary Table S6. Association of study arm with home-level vaccination uptake by race

Supplementary Table S6A. Non-White Residents: Association of study arm with vaccination uptake (n=374, subgroup analysis)

Fixed effects	HR	SE	95% CI	p-value
Intervention effect	2.25	0.25	(1.38, 3.68)	0.001
Stratum 1	Ref	-	-	-
Stratum 2	2.30	0.60	(0.71, 7.42)	0.16
Stratum 3	1.38	1.14	(0.15, 12.98)	0.78
Stratum 4	0.00	9459.19	(0.00, Inf)	> 0.99[a]
Stratum 5	0.32	0.61	(0.10, 1.06)	0.06
Stratum 6	0.30	0.57	(0.10, 0.92)	0.04
Stratum 7	0.13	0.71	(0.03, 0.53)	0.004
Stratum 8	0.39	0.58	(0.12, 1.23)	0.11
Agency 1	1.32	0.57	(0.43, 4.03)	0.63
Agency 2	2.76	0.32	(1.47, 5.18)	0.002
Agency 3	0.85	0.52	(0.31, 2.32)	0.74
Agency 4	8.61	0.47	(3.44, 21.59)	< 0.001
Agency 5	0.00	10423.07	(0.00, Inf)	> 0.99[a]
Agency 6	Ref	-	-	-

[a]Only 5 participants in this category, so did not converge

Supplementary Table S6B. Non-Hispanic White Residents: Association of study arm with vaccination uptake (n=461, subgroup analysis)

Fixed effects	HR	SE	95% CI	p-value
Intervention effect	0.79	0.35	(0.40, 1.55)	0.49
Stratum 1	Ref	-	-	-
Stratum 2	2.42	1.09	(0.29, 20.35)	0.42
Stratum 3	2.91	0.99	(0.42, 20.09)	0.28
Stratum 4	1.43	1.05	(0.18, 11.13)	0.73
Stratum 5	0.42	1.15	(0.04, 4.05)	0.46
Stratum 6	0.81	0.94	(0.13, 5.13)	0.82
Stratum 7	0.90	0.94	(0.14, 5.72)	0.91
Stratum 8	0.48	0.94	(0.08, 3.00)	0.43
Agency 1	2.68	0.54	(0.92, 7.79)	0.07
Agency 2	3.58	0.60	(1.10, 11.64)	0.03
Agency 3	3.30	0.69	(0.85, 12.78)	0.08
Agency 4	3.21	0.57	(1.05, 9.81)	0.04
Agency 5	5.06	0.67	(1.35, 18.90)	0.02
Agency 6	Ref	-	-	-

Supplementary Table S6C. Residents: Treatment effect heterogeneity on vaccine uptake by race 4/1/2021–6/30/2022 (n= 899)

Fixed effects	HR	SE	95% CI	p-value
Intervention effect	0.83	0.29	(0.47, 1.47)	0.52
Stratum 1	Ref	-	-	-
Stratum 2	2.48	0.60	(0.77, 7.98)	0.13
Stratum 3	1.79	0.64	(0.51, 6.24)	0.36
Stratum 4	1.03	0.71	(0.26, 4.11)	0.97
Stratum 5	0.35	0.61	(0.11, 1.13)	0.08
Stratum 6	0.51	0.56	(0.17, 1.53)	0.23
Stratum 7	0.53	0.59	(0.17, 1.67)	0.28
Stratum 8	0.49	0.56	(0.16, 1.46)	0.20
Agency 1	2.06	0.38	(0.99, 4.31)	0.05
Agency 2	3.47	0.31	(1.89, 6.34)	< 0.001
Agency 3	2.40	0.42	(1.05, 5.49)	0.04
Agency 4	3.82	0.36	(1.88, 7.75)	< 0.001
Agency 5	3.38	0.52	(1.22, 9.35)	0.02
Agency 6	Ref	-	-	-
White	Ref	-	-	-
Non-White	0.98	0.30	(0.54, 1.76)	0.93
Missing race	1.36	0.47	(0.54, 3.44)	0.51
Intervention arm x non-white race	2.28	0.39	(1.07, 4.87)	0.03
Intervention arm x missing race	0.81	0.79	(0.17, 3.83)	0.79

Supplementary Table S6D. Staff: Treatment effect heterogeneity on vaccine uptake by race between 4/1/2021-6/30/2022 (n = 938)

Fixed effects	HR	SE	95% CI	p-value
Intervention effect	0.82	0.20	(0.56, 1.21)	0.32
Stratum 1	Ref	-	-	-
Stratum 2	1.21	0.13	(0.93, 1.57)	0.15
Stratum 3	1.14	0.16	(0.83, 1.55)	0.41
Stratum 4	0.82	0.15	(0.61, 1.12)	0.21
Stratum 5	1.29	0.16	(0.95, 1.76)	0.10
Stratum 6	1.19	0.14	(0.90, 1.58)	0.21
Stratum 7	0.74	0.20	(0.50, 1.09)	0.12
Stratum 8	1.42	0.15	(1.06, 1.91)	0.02
Agency 1	1.37	0.11	(1.10, 1.70)	0.005
Agency 2	0.59	0.11	(0.48, 0.74)	< 0.001
Agency 3	0.96	0.12	(0.76, 1.22)	0.76
Agency 4	0.39	0.18	(0.28, 0.55)	< 0.001
Agency 5	1.22	0.14	(0.93, 1.61)	0.15
Agency 6	Ref	-	-	-
White	Ref	-	-	-
Non-White	1.20	0.15	(0.90, 1.59)	0.22
Missing race	0.94	0.25	(0.58, 1.55)	0.82
Intervention arm x non-white race	1.25	0.21	(0.83, 1.89)	0.29
Intervention arm x missing race	1.25	0.37	(0.61, 2.57)	0.54

Supplementary Table S7. Association of study arm with home-level vaccination uptake among residents by group home type

Supplementary Table S7A. Residents with ID/DD: Association of study arm with home-level time to vaccination between 1/2021–7/2022 (n=123)

Fixed effects	HR	SE	p-value	95% CI
Intervention arm	1.76	0.60	0.34	(0.55, 5.66)
Stratum 2	4.55	1.12	0.17	(0.51, 40.46)
Stratum 3	1.55	1.10	0.69	(0.18, 13.43)
Stratum 4	1.19	1.16	0.88	(0.12, 11.48)
Agency 2	0.51	1.07	0.53	(0.06, 4.22)
Agency 3	16.56	0.97	0.004	(2.46, 111.67)
Agency 4	1.81	1.10	0.59	(0.21, 15.65)
Agency 5	5.56	1.15	0.14	(0.58, 52.96)
Agency 6	224.02	2.09	0.01	(3.76, 13352.45)

Supplementary Table S7B. Residents with SMI: Association of study arm with home-level time to vaccination between 1/2021–7/2022 (n=776)

Fixed effects	HR	SE	p-value	95% CI
Intervention arm	1.07	0.22	0.78	(0.69, 1.65)
Stratum 6	1.37	0.34	0.36	(0.70, 2.68)
Stratum 7	1.30	0.47	0.57	(0.52, 3.24)
Stratum 8	1.11	0.39	0.78	(0.52, 2.37)
Agency 2	3.80	0.40	< 0.001	(1.74, 8.28)
Agency 3	3.87	0.33	< 0.001	(2.02, 7.41)
Agency 4	3.96	0.47	0.004	(1.57, 9.96)
Agency 5	4.53	0.41	< 0.001	(2.04, 10.10)
Agency 6	2.94	0.51	0.03	(1.09, 7.92)

Supplementary Table S8. Primary Implementation Outcome: COVID-19 Best Practices Fidelity

Supplementary Table S8A. Association of GBP and TBP practices with Fidelity scores (n=296)

Fixed effects	Estimate	SE	*p*-val	95% CI		
Stratum 2[a]	-0.53	1.30	0.68	(-3.07, 2.01)		
Stratum 3	0.82	1.44	0.57	(-2.00, 3.64)		
Stratum 4	-1.11	1.39	0.43	(-3.84, 1.62)		
Stratum 5	-7.30	1.37	< 0.001	(-9.98, -4.61)		
Stratum 6	-7.00	1.40	< 0.001	(-9.76, -4.25)		
Stratum 7	-6.26	1.36	< 0.001	(-8.93, -3.59)		
Stratum 8	-6.85	1.41	< 0.001	(-9.62, -4.09)		
Agency 1[b]	1.79	0.97	0.07	(-0.12, 3.69)		
Agency 2	-1.33	0.94	0.16	(-3.17, 0.51)		
Agency 3	1.49	1.28	0.24	(-1.02, 4.00)		
Agency 4	0.22	1.37	0.87	(-2.46, 2.89)		
Agency 5	-1.08	1.28	0.40	(-3.59, 2.89)		
Baseline home-level score	1.79	0.97	0.07	(-0.12, 3.69)		
Linear time trend	0.03	0.35	0.92	(-0.64, 0.71)		
Quadratic time trend (time^2)	0.00	0.02	0.84	(-0.04, 0.03)		
					**Joint test for intervention effect**[c]	
Fixed effects	**Estimate**	**SE**	***p*-val**	**95% CI**	**Test statistic**	***p*-val**
Intervention arm main effect	1.51	1.92	0.43	(-2.24, 5.26)	3.12	>.99
Intervention x time interaction	-0.05	0.47	0.92	(-0.98, 0.88)		
Intervention x time^2 interaction	0.00	0.03	0.98	(-0.05, 0.05)		
Variance component	18.30	2.51		(13.98, 23.95)		

Abbreviation: GBP, General Best Practice; TBP, Tailored Best Practice; SE, Standard error; CI, Confidence interval
[a]Stratification factors used for randomization

[b] Agency name and identifiers suppressed to protect the confidentiality of health-related outcomes. Agency 6 was the reference.
[c] Joint Wald test was performed to assess whether there were significant differences in the trends between treatment groups. Pvalue was adjusted for multiple comparisons using the Bonferroni correction.

Supplementary Table S8B. Group-home-specific fidelity scores by time point

	Raw Score		Marginal mean Score			Unadjusted
Time period	GBP	TBP	GBP	TBP	Mean Difference	95% CI
1-3 months	86.45	86.47	85.77	87.14	1.37	(-0.51, 3.25)
4-6 months	85.49	86.21	85.30	86.54	1.24	(-0.24, 2.73)
7-9 months	85.14	86.16	84.89	86.02	1.13	(-0.49, 2.74)
10-12 months	85.72	85.35	85.15	86.17	1.02	(-0.48, 2.52)
13-15 months	85.38	85.56	84.81	85.73	0.93	(-1.00, 2.86)

*Marginal mean scores and mean difference between scores are post-hoc analyses based on the previous model.

Supplementary Table S9. Acceptability

Supplementary Table S9A. Association of GBP and TBP with group home-level Acceptability scores (n=278)

Fixed effects	Estimate	SE	*p*-val	95% CI		
Stratum 2[a]	-0.09	0.11	0.42	(-0.31, 0.13)		
Stratum 3	-0.05	0.12	0.66	(-0.30, 0.19)		
Stratum 4	-0.11	0.12	0.34	(-0.35, 0.12)		
Stratum 5	-0.29	0.12	0.02	(-0.53, -0.05)		
Stratum 6	-0.06	0.12	0.58	(-0.30, 0.17)		
Stratum 7	-0.37	0.12	0.002	(-0.60, -0.13)		
Stratum 8	-0.25	0.12	0.03	(-0.49, -0.02)		
Agency 1[b]	0.04	0.09	0.62	(-0.13, 0.21)		
Agency 2	0.03	0.08	0.67	(-0.13, 0.20)		
Agency 3	0.20	0.11	0.07	(-0.02, 0.42)		
Agency 4	-0.21	0.12	0.08	(-0.44, 0.42)		
Agency 5	-0.12	0.11	0.28	(-0.34, 0.10)		
Baseline home-level score	0.24	0.04	< 0.001	(0.16, 0.31)		
Linear time trend	0.02	0.03	0.50	(-0.04, 0.08)		
Quadratic time trend (time^2)	0.00	0.00	0.38	(0.00, 0.00)		
					**Joint test for intervention effect**[c]	
Fixed effects	**Estimate**	**SE**	***p*-val**	**95% CI**	**Test statistic**	***p*-val**
Intervention arm main effect	0.01	0.16	0.95	(-0.30, 0.32)	11.28	0.01
Intervention x time interaction	0.04	0.04	0.35	(-0.04, 0.11)		
Intervention x time^2 interaction	0.00	0.00	0.47	(-0.01, 0.00)		
Variance component	0.14	0.02		(0.11, 0.19)		

Abbreviation: GBP, General Best Practice; TBP, Tailored Best Practice; SE, Standard error; CI, Confidence interval
[a] Stratification factors used for randomization
[b] Agency name and identifiers suppressed to protect the confidentiality of health-related outcomes
[c] Joint Wald test was performed to assess whether there were significant differences in the trends between treatment groups

Supplementary Table S9B. Group-home-specific Acceptability scores by time point

	Raw Score		Marginal mean Score			Unadjusted
Time period	GBP	TBP	GBP	TBP	Mean Difference	95% CI
1-3 months	3.99	4.09	3.99	4.10	0.10	(-0.06, 0.26)
4-6 months	4.10	4.12	4.02	4.19	0.17	(0.04, 0.30)
7-9 months	4.03	4.19	4.00	4.21	0.21	(0.07, 0.35)
10-12 months	3.98	4.20	3.97	4.19	0.22	(0.09, 0.35)
13-15 months	3.99	4.07	3.92	4.12	0.20	(0.04, 0.37)

*Marginal mean scores and mean difference between scores are post-hoc analyses based on the previous model.

Supplementary Figure 9C. Marginal mean Acceptability scores by study arm (zoomed scale) (n=278)

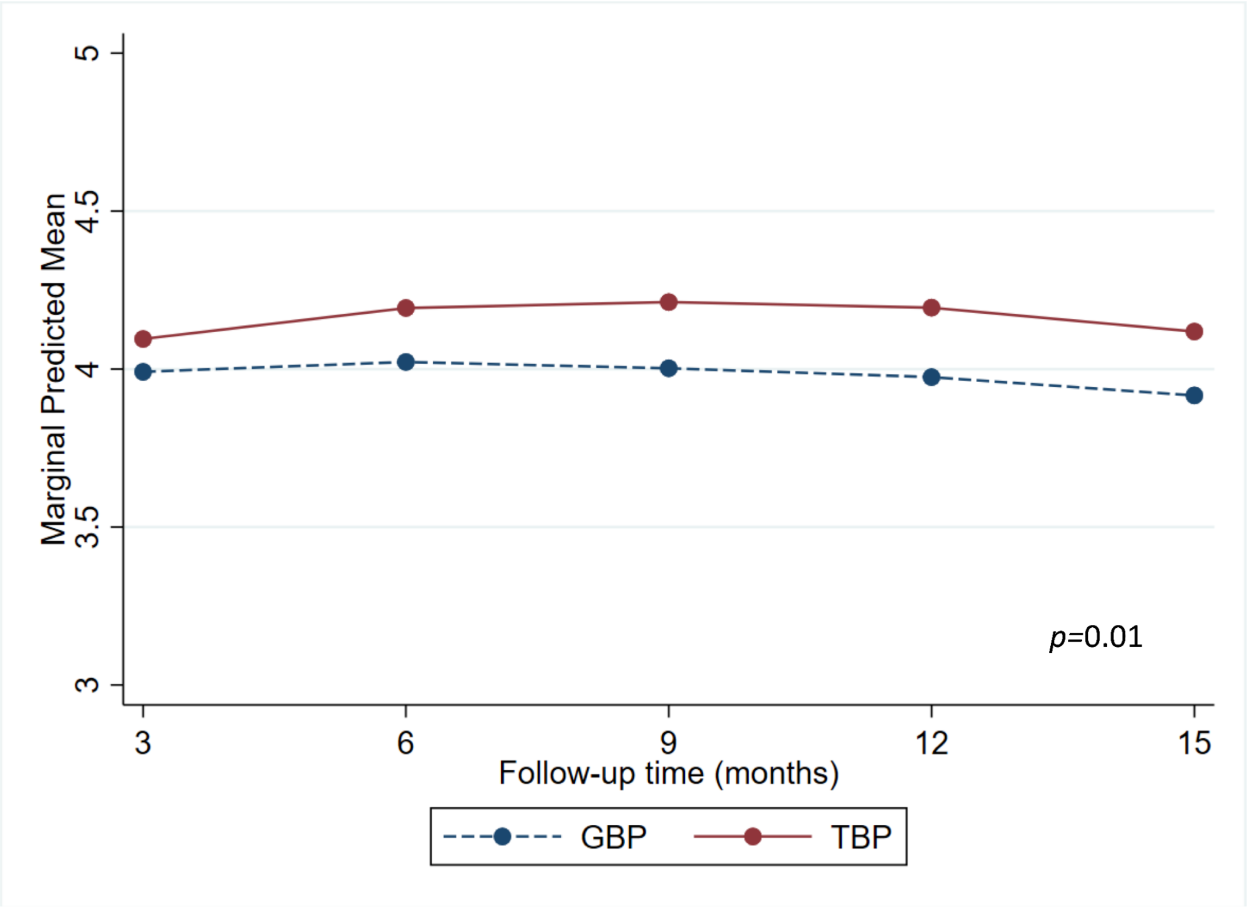

Note: Month ranges represented on x-axis: 3, Apr '21 – Jun '21; 6, Jul '21 – Sep '21; 9, Oct '21 – Dec '21; 12, Jan'22 – Mar'22; 15, Apr '22 – Jun-22

Supplementary Table S10. Appropriateness

Supplementary Table S10A. Association of GBP and TBP with group home-level Appropriateness (n=279)

Fixed effects	Estimate	SE	*p*-val	95% CI		
Stratum 2[a]	0.02	0.11	0.88	(-0.20, 0.23)		
Stratum 3	-0.01	0.12	0.94	(-0.24, 0.23)		
Stratum 4	-0.03	0.12	0.82	(-0.25, 0.20)		
Stratum 5	-0.30	0.12	0.01	(-0.53, -0.07)		
Stratum 6	-0.16	0.11	0.17	(-0.38, 0.07)		
Stratum 7	-0.41	0.12	< 0.001	(-0.64, -0.19)		
Stratum 8	-0.42	0.12	< 0.001	(-0.65, -0.19)		
Agency 1[b]	-0.03	0.08	0.76	(-0.19, 0.14)		
Agency 2	0.06	0.08	0.48	(-0.10, 0.21)		
Agency 3	0.25	0.11	0.02	(0.04, 0.46)		
Agency 4	-0.20	0.12	0.08	(-0.43, 0.46)		
Agency 5	-0.07	0.11	0.54	(-0.28, 0.15)		
Baseline home-level score	0.24	0.03	< 0.001	(0.17, 0.30)		
Linear time trend	-0.01	0.03	0.81	(-0.06, 0.05)		
Quadratic time trend (time^2)	0.00	0.00	0.97	(0.00, 0.00)		
					**Joint test for intervention effect**[c]	
Fixed effects	**Estimate**	**SE**	***p*-val**	**95% CI**	**Test statistic**	***p*-val**
Intervention arm main effect	-0.16	0.16	0.31	(-0.48, 0.15)	12.74	0.005
Intervention x time interaction	0.04	0.04	0.28	(-0.03, 0.12)		
Intervention x time^2 interaction	0.00	0.00	0.60	(-0.01, 0.00)		
Variance component	0.13	0.02		(0.10, 0.17)		

Abbreviation: GBP, General Best Practice; TBP, Tailored Best Practice; SE, Standard error; CI, Confidence interval
[a] Stratification factors used for randomization
[b] Agency name and identifiers suppressed to protect the confidentiality of health-related outcomes
[c] Joint Wald test was performed to assess whether there were significant differences in the trends between treatment groups

Supplementary Table S10B. Group-home-specific staff Appropriateness scores by time point

	Raw Score		Marginal mean Score		Unadjusted	
Time period	**GBP**	**TBP**	**GBP**	**TBP**	**Mean Difference**	**95% CI**
1-3 months	3.95	3.94	3.97	3.93	-0.04	(-0.20, 0.11)
4-6 months	4.03	3.93	3.96	4.01	0.05	(-0.07, 0.18)
7-9 months	3.83	4.05	3.92	4.05	0.13	(0.00, 0.27)
10-12 months	3.97	4.13	3.91	4.09	0.19	(0.06, 0.31)
13-15 months	3.89	4.07	3.88	4.11	0.23	(0.06, 0.39)

*Marginal mean scores and mean difference between scores are post-hoc analyses based on the previous model.

Supplementary Figure S10C. Marginal mean Appropriateness scores by study arm (zoomed scale) (n=279)

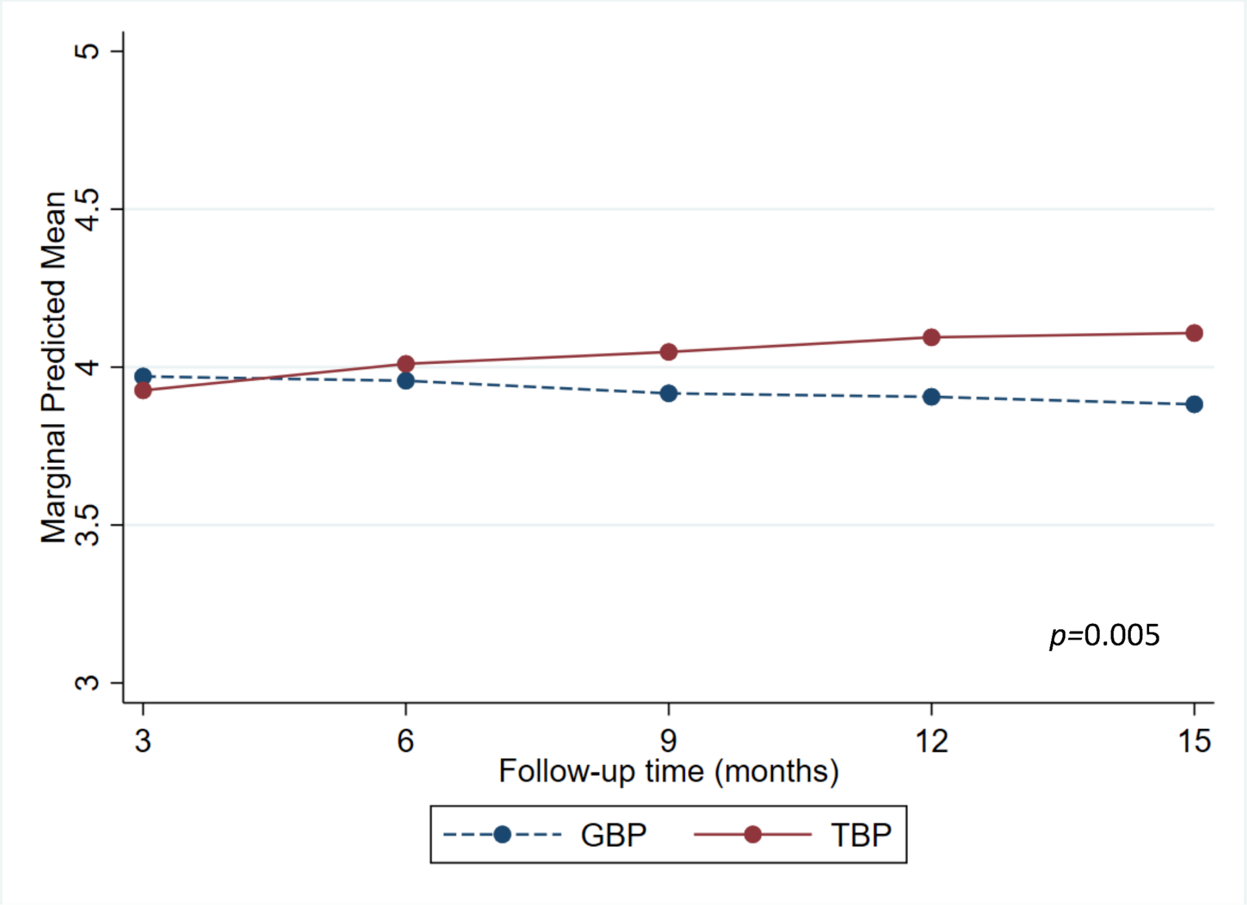

Note: Month ranges represented on x-axis: 3, Apr '21 – Jun '21; 6, Jul ‘21 – Sep ‘21; 9, Oct ‘21 – Dec ‘21; 12, Jan’22 – Mar’22; 15, Apr ‘22 – Jun-22

Supplementary Table S11. Feasibility

Supplementary Table S11A. Association of GBP and TBP with group home-level Feasibility (n=278)

Fixed effects	Estimate	SE	*p*-val	95% CI		
Stratum 2[a]	-0.01	0.10	0.95	(-0.21, 0.19)		
Stratum 3	-0.13	0.11	0.25	(-0.35, 0.09)		
Stratum 4	-0.08	0.11	0.45	(-0.29, 0.13)		
Stratum 5	-0.26	0.11	0.02	(-0.47, -0.04)		
Stratum 6	-0.17	0.11	0.11	(-0.38, 0.04)		
Stratum 7	-0.34	0.11	0.001	(-0.55, -0.14)		
Stratum 8	-0.35	0.11	0.01	(-0.55, -0.14)		
Agency 1[b]	-0.02	0.08	0.76	(-0.17, 0.13)		
Agency 2	0.06	0.07	0.46	(-0.09, 0.20)		
Agency 3	0.19	0.10	0.07	(-0.01, 0.38)		
Agency 4	-0.11	0.11	0.31	(-0.32, 0.38)		
Agency 5	-0.08	0.10	0.44	(-0.27, 0.12)		
Baseline home-level score	0.21	0.04	< 0.001	(0.14, 0.29)		
Linear time trend	0.04	0.03	0.15	(-0.01, 0.09)		
Quadratic time trend (time^2)	0.00	0.00	0.14	(0.00, 0.00)		
					**Joint test for intervention effect**[c]	
Fixed effects	**Estimate**	**SE**	***p*-val**	**95% CI**	**Test statistic**	***p*-val**
Intervention arm main effect	0.04	0.14	0.77	(-0.24, 0.32)	13.49	0.004
Intervention x time interaction	-0.01	0.04	0.86	(-0.08, 0.06)		
Intervention x time^2 interaction	0.00	0.00	0.48	(0.00, 0.01)		
Variance component	0.12	0.02		(0.09, 0.15)		

Abbreviation: GBP, General Best Practice; TBP, Tailored Best Practice; SE, Standard error; CI, Confidence interval
[b] Stratification factors used for randomization
[b] Agency name and identifiers suppressed to protect the confidentiality of health-related outcomes
[c] Joint Wald test was performed to assess whether there were significant differences in the trends between treatment groups

Supplementary Table S11B. Group-home-specific staff Feasibility scores by time point

	Raw Score		Marginal mean Score			Unadjusted
Time period	GBP	TBP	GBP	TBP	Mean Difference	95% CI
1-3 months	4.17	4.18	4.02	4.05	0.04	(-0.11, 0.18)
4-6 months	4.22	4.17	4.08	4.13	0.05	(-0.06, 0.17)
7-9 months	4.19	4.32	4.09	4.19	0.10	(-0.02, 0.22)
10-12 months	4.26	4.37	4.06	4.23	0.17	(0.05, 0.28)
13-15 months	4.14	4.35	4.00	4.26	0.26	(0.11, 0.41)

*Marginal mean scores and mean difference between scores are post-hoc analyses based on the previous model.

Supplementary Figure 11C. Marginal mean Feasibility scores by study arm (zoomed scale) (n=278)

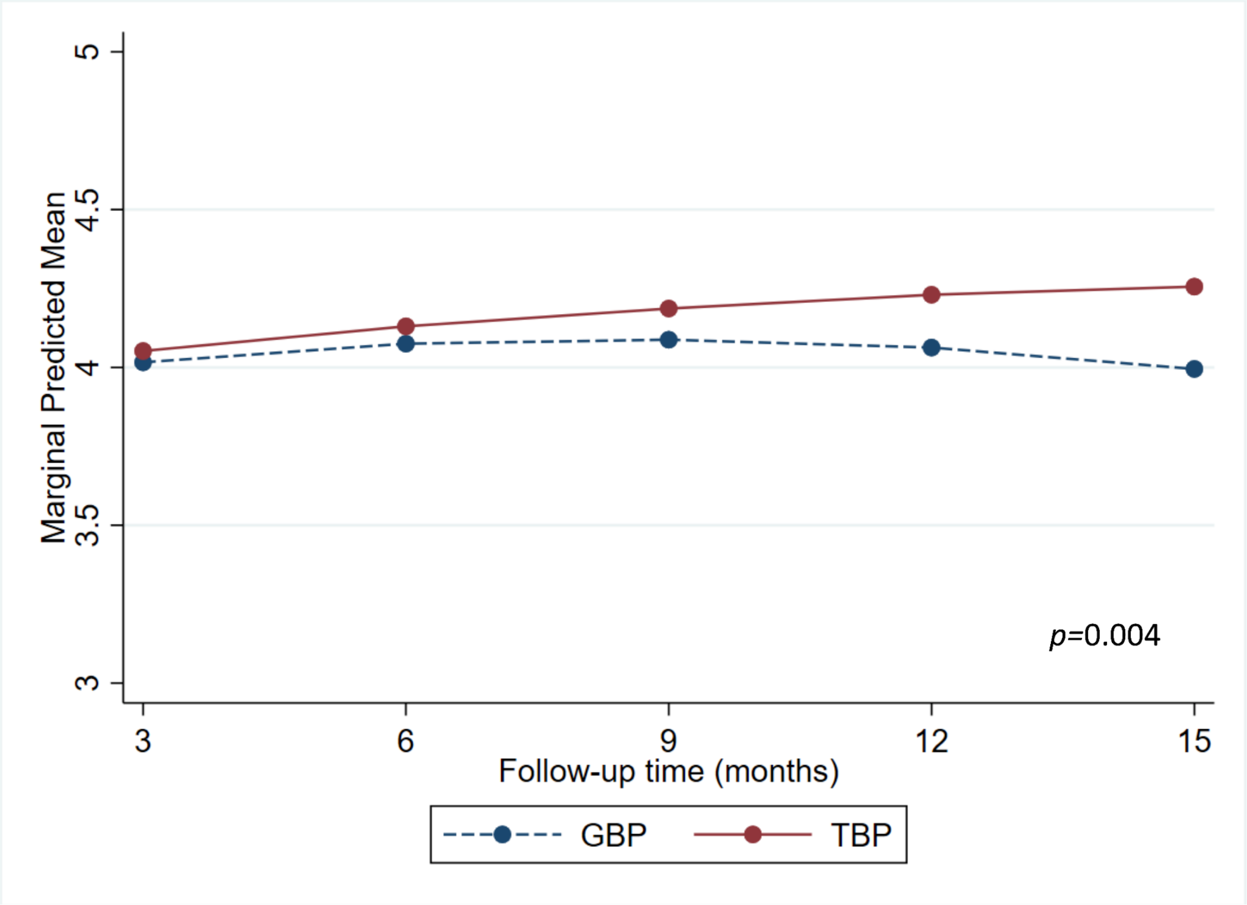

Note: Month ranges represented on x-axis: 3, Apr '21 – Jun '21; 6, Jul '21 – Sep '21; 9, Oct '21 – Dec '21; 12, Jan'22 – Mar'22; 15, Apr '22 – Jun-22

Supplementary Table S12. Reach

Supplementary Table S12A. Tailored Best Practices Implementation Reach

Fixed effects		**Goal count of activities**	**Count of activities completed**	**Proportion of homes that received HP**
By population:	Overall[a]	942	914	97%
	Staff	416	408	98%
	Residents	318	293	92%
By Intervention activity:	Motivational interviews	313	304	97%
	Trusted messenger	318	308	97%
	Interactive education	103	90	87%

[a] Includes Initial House Plan, Motivational interviews, Trusted Messenger, Interactive Education

Supplementary Table S12B. Proportion of Group homes with complete and partial intervention completion by agency

Fixed effects	**100% of intervention activities completed**	**Partial intervention activities completed**	**0% intervention activities completed**
Agency 1	77%	23%	0%
Agency 2	95%	5%	0%
Agency 3	100%	0%	0%
Agency 4	87%	13%	0%
Agency 5	93%	7%	0%
Agency 6	91%	9%	0%
Total:	90%	10%	0%

Supplementary Table S13. Primary Effectiveness Outcome: Associations of GBP and TBP with group home-level COVID-19 incidence by time point

Supplementary Table S13A. Residents and staff combined: Associations of GBP and TBP with group home-level COVID-19 incidence (n=415)

Fixed effects	Estimate	SE	*p*-val	95% CI		
Stratum 2[a]	0.97	0.09	0.73	(0.81, 1.16)		
Stratum 3	0.87	0.09	0.16	(0.72, 1.06)		
Stratum 4	0.96	0.10	0.69	(0.79, 1.17)		
Stratum 5	0.72	0.07	0.001	(0.59, 0.88)		
Stratum 6	0.75	0.07	0.003	(0.62, 0.91)		
Stratum 7	0.67	0.07	< 0.001	(0.55, 0.82)		
Stratum 8	0.72	0.07	0.001	(0.59, 0.87)		
Agency 1[b]	0.74	0.06	< 0.001	(0.64, 0.86)		
Agency 2	0.55	0.04	< 0.001	(0.47, 0.64)		
Agency 3	1.13	0.09	0.14	(0.96, 1.33)		
Agency 4	1.22	0.10	0.02	(1.03, 1.33)		
Agency 5	0.93	0.09	0.42	(0.77, 1.11)		
Baseline home-level incidence	1.00	0.01	0.75	(0.98, 1.02)		
Linear time trend	4.07	0.30	< 0.001	(3.52, 4.69)		
Quadratic time trend (time^2)	0.94	0.00	< 0.001	(0.94, 0.95)		
					**Joint test for intervention effect**[c]	
Fixed effects	**Estimate**	**SE**	***p*-val**	**95% CI**	**Test statistic**	***p*-val**
Intervention arm main effect	0.33	0.20	0.06	(0.10, 1.07)	5.34	0.89
Intervention x time interaction	1.20	0.13	0.10	(0.97, 1.49)		
Intervention x time^2 interaction	0.99	0.00	0.15	(0.98, 1.00)		
Variance component	0.06	0.02		(0.04, 0.10)		

Abbreviation: GBP, General Best Practice; TBP, Tailored Best Practice; SE, Standard error; CI, Confidence interval
[a] Stratification factors used for randomization
[b] Agency name and identifiers suppressed to protect the confidentiality of health-related outcomes
[c] Joint Wald test was performed to assess whether there were significant differences in the trends between treatment groups. Pvalue was adjusted for multiple comparisons using the Bonferroni correction.

Supplementary Table S13B. Residents and staff combined: Associations of GBP and TBP with group home-level COVID-19 incidence by time point

	Raw incidence rate[a]		Marginal mean incidence rate		Unadjusted	
Time period	**GBP**	**TBP**	**GBP**	**TBP**	**IRR**	**95% CI**
1-3 months[b]	0.16	0.09	0.05	0.03	0.54	(0.29, 1.00)
4-6 months	0.69	0.55	0.66	0.50	0.77	(0.59, 1.00)
7-9 months	2.27	2.35	2.97	2.87	0.97	(0.86, 1.10)
10-12 months	5.67	5.75	4.56	4.90	1.08	(0.96, 1.21)
13-15 months	2.22	2.35	2.38	2.50	1.06	(0.89, 1.25)

Abbreviation: GBP, General Best Practice; TBP, Tailored Best Practice; IRR, Incident rate ratio; CI, Confidence interval
[a] Raw and marginal mean COVID-19 incidence rate per 100 person-months
[b] Where month 1 is January, 2021.

Supplementary Table S13C. Staff: Associations of GBP and TBP with group home-level COVID-19 incidence (n=415)

Fixed effects	Estimate	SE	*p*-val	95% CI		
Stratum 2[a]	1.05	0.09	0.60	(0.88, 1.24)		
Stratum 3	0.92	0.09	0.37	(0.76, 1.11)		
Stratum 4	1.10	0.10	0.32	(0.91, 1.33)		
Stratum 5	1.01	0.10	0.93	(0.84, 1.22)		
Stratum 6	0.92	0.09	0.41	(0.77, 1.11)		
Stratum 7	0.89	0.09	0.23	(0.73, 1.08)		
Stratum 8	0.98	0.09	0.87	(0.82, 1.19)		
Agency 1[b]	0.78	0.06	< 0.001	(0.68, 0.90)		
Agency 2	0.51	0.04	< 0.001	(0.43, 0.59)		
Agency 3	1.23	0.09	0.005	(1.07, 1.43)		
Agency 4	1.27	0.11	0.01	(1.07, 1.43)		
Agency 5	0.94	0.08	0.46	(0.79, 1.11)		
Baseline home-level incidence	1.01	0.01	0.20	(0.99, 1.03)		
Linear time trend	4.12	0.34	< 0.001	(3.50, 4.85)		
Quadratic time trend (time^2)	0.94	0.00	< 0.001	(0.93, 0.95)		
					Joint test for intervention effect[c]	
Fixed effects	Estimate	SE	*p*-val	95% CI	Test statistic	*p*-val
Intervention arm main effect	0.39	0.26	0.16	(0.11, 1.46)	2.27	>.99
Intervention x time interaction	1.18	0.15	0.18	(0.93, 1.51)		
Intervention x time^2 interaction	0.99	0.01	0.19	(0.98, 1.00)		
Variance component	0.00	0.00		(0.00, 0.00)		

Abbreviation: GBP, General Best Practice; TBP, Tailored Best Practice; SE, Standard error; CI, Confidence interval
[a] Stratification factors used for randomization
[b] Agency name and identifiers suppressed to protect the confidentiality of health-related outcomes
[c] Joint Wald test was performed to assess whether there were significant differences in the trends between treatment groups Pvalue was adjusted for multiple comparisons using the Bonferroni correction.

Supplementary Table S13D. Staff: Associations of GBP and TBP with group home-level COVID-19 incidence by time point

	Raw incidence rate[a]		Marginal mean incidence rate		Unadjusted	
Time period	**GBP**	**TBP**	**GBP**	**TBP**	**IRR**	**95% CI**
1-3 months[b]	0.27	0.14	0.06	0.04	0.61	(0.30, 1.22)
4-6 months	0.64	0.66	0.79	0.65	0.82	(0.62, 1.10)
7-9 months	2.90	2.87	3.65	3.57	0.98	(0.86, 1.11)
10-12 months	6.94	6.86	5.65	5.74	1.02	(0.91, 1.14)
13-15 months	2.85	2.40	2.94	2.72	0.93	(0.77, 1.12)

Abbreviation: GBP, General Best Practice; TBP, Tailored Best Practice; IRR, Incident rate ratio; CI, Confidence interval
[a] Raw and marginal mean COVID-19 incidence rate per 100 person-months
[c] Where month 1 is January, 2021.

Supplementary Table S13E. Resident (ID/DD and SMI combined): Associations of GBP and TBP with group home-level COVID-19 incidence (n=415)

Fixed effects	Estimate	SE	*p*-val	95% CI		
Stratum 2[a]	0.78	0.19	0.30	(0.48, 1.25)		
Stratum 3	0.75	0.18	0.24	(0.46, 1.21)		
Stratum 4	0.58	0.16	0.05	(0.34, 1.00)		
Stratum 5	0.28	0.08	< 0.001	(0.17, 0.48)		
Stratum 6	0.53	0.13	0.01	(0.33, 0.85)		
Stratum 7	0.42	0.10	0.001	(0.26, 0.69)		
Stratum 8	0.42	0.10	0.001	(0.25, 0.68)		
Agency 1[b]	0.46	0.10	< 0.001	(0.30, 0.70)		
Agency 2	0.63	0.11	0.01	(0.45, 0.90)		
Agency 3	0.49	0.13	0.01	(0.29, 0.81)		
Agency 4	1.27	0.25	0.22	(0.86, 0.81)		
Agency 5	0.94	0.23	0.80	(0.58, 1.51)		
Baseline home-level incidence	0.99	0.01	0.67	(0.97, 1.02)		
Linear time trend	4.01	0.63	< 0.001	(2.95, 5.45)		
Quadratic time trend (time^2)	0.94	0.01	< 0.001	(0.93, 0.96)		
					**Joint test for intervention effect**[b]	
Fixed effects	**Estimate**	**SE**	***p*-val**	**95% CI**	**Test statistic**	***p*-val**
Intervention arm main effect	0.08	0.11	0.07	(0.00, 1.20)	10.50	0.09
Intervention x time interaction	1.46	0.37	0.13	(0.89, 2.38)		
Intervention x time^2 interaction	0.99	0.01	0.26	(0.97, 1.01)		
Variance component	0.59	0.11		(0.41, 0.86)		

Abbreviation: GBP, General Best Practice; TBP, Tailored Best Practice; SE, Standard error; CI, Confidence interval
[a] Stratification factors used for randomization
[b] Joint Wald test was performed to assess whether there were significant differences in the trends between treatment groups. Pvalue was adjusted for multiple comparisons using the Bonferroni correction.

Supplementary Table S13F. Resident (ID/DD and SMI combined): Associations of GBP and TBP with group home-level COVID-19 incidence by time point

	Raw incidence rate[a]		Marginal mean incidence rate		Unadjusted	
Time period	**GBP**	**TBP**	**GBP**	**TBP**	**IRR**	**95% CI**
1-3 months[b]	0.00	0.00	0.03	0.01	0.21	(0.05, 0.96)
4-6 months	0.71	0.39	0.41	0.19	0.48	(0.25, 0.92)
7-9 months	1.50	1.31	1.85	1.51	0.85	(0.63, 1.15)
10-12 months	3.91	4.20	2.86	3.32	1.22	(0.93, 1.60)
13-15 months	1.91	2.26	1.52	2.02	1.40	(0.98, 1.99)

Abbreviation: GBP, General Best Practice; TBP, Tailored Best Practice; IRR, Incident rate ratio; CI, Confidence interval
[a] Raw and marginal mean COVID-19 incidence rate per 100 person-months
[c] Where month 1 is January, 2021.

Supplementary Table S13G. Residents with SMI: Associations of GBP and TBP with group home-level COVID-19 incidence (n=209)

Fixed effects	**Estimate**	**SE**	***p*-val**	**95% CI**		
Stratum 6[a]	1.71	0.46	0.05	(1.01, 2.90)		
Stratum 7	2.05	0.61	0.02	(1.15, 3.67)		
Stratum 8	1.59	0.46	0.11	(0.90, 2.79)		
Agency 1[b]	0.81	0.25	0.50	(0.44, 1.49)		
Agency 2	1.01	0.25	0.97	(0.62, 1.65)		
Agency 3	0.89	0.33	0.76	(0.44, 1.83)		
Agency 4	0.91	0.26	0.74	(0.51, 1.61)		
Agency 5	0.75	0.30	0.47	(0.34, 1.64)		
Baseline home-level incidence	0.95	0.04	0.16	(0.88, 1.02)		
Linear time trend	6.44	1.72	< 0.001	(3.81, 10.87)		
Quadratic time trend (time^2)	0.92	0.01	< 0.001	(0.90, 0.00)		
					**Joint test for intervention effect**[c]	
Fixed effects	**Estimate**	**SE**	***p*-val**	**95% CI**	**Test statistic**	***p*-val**
Intervention arm main effect	0.19	0.43	0.46	(0.00, 14.72)	14.75	0.002
Intervention x time interaction	1.18	0.47	0.67	(0.55, 2.56)		
Intervention x time^2 interaction	1.00	0.02	0.99	(0.97, 1.03)		
Variance component	0.68	0.17		(0.42, 1.11)		

Abbreviation: GBP, General Best Practice; TBP, Tailored Best Practice; SE, Standard error; CI, Confidence interval
[a] Stratification factors used for randomization
[b] Agency name and identifiers suppressed to protect the confidentiality of health-related outcomes
[c] Joint Wald test was performed to assess whether there were significant differences in the trends between treatment groups

Supplementary Table S13H. Residents with SMI: Associations of GBP and TBP with group home-level COVID-19 incidence by time point

	Raw incidence rate[a]		Marginal mean incidence rate		Unadjusted	
Time period	**GBP**	**TBP**	**GBP**	**TBP**	**IRR**	**95% CI**
1-3 months[b]	0.00	0.00	0.01	0.00	0.32	(0.03, 3.39)
4-6 months	0.35	0.26	0.23	0.10	0.54	(0.19, 1.49)
7-9 months	1.16	1.10	1.45	1.09	0.90	(0.58, 1.40)
10-12 months	2.71	3.59	2.06	2.62	1.52	(1.03, 2.24)
13-15 months	0.78	1.60	0.66	1.41	2.57	(1.45, 4.55)

Abbreviation: GBP, General Best Practice; TBP, Tailored Best Practice; IRR, Incident rate ratio; CI, Confidence interval
[a] Raw and marginal mean COVID-19 incidence rate per 100 person-months
[b] Where month 1 is January, 2021.

Supplementary Table S13I. Residents with ID/DD: Associations of GBP and TBP with group home-level COVID-19 incidence (n=206)

Fixed effects	Estimate	SE	*p*-val	95% CI		
Stratum 2[a]	0.82	0.18	0.38	(0.54, 1.26)		
Stratum 3	0.70	0.16	0.12	(0.45, 1.10)		
Stratum 4	0.63	0.15	0.06	(0.39, 1.01)		
Agency 1[b]	0.29	0.08	< 0.001	(0.17, 0.50)		
Agency 2	0.46	0.12	0.002	(0.28, 0.75)		
Agency 3	0.31	0.11	0.001	(0.16, 0.62)		
Agency 4	1.53	0.39	0.09	(0.93, 2.51)		
Agency 5	1.09	0.30	0.75	(0.64, 1.87)		
Baseline home-level incidence	1.00	0.01	0.90	(0.97, 1.02)		
Linear time trend	2.92	0.57	< 0.001	(1.99, 4.27)		
Quadratic time trend (time^2)	0.96	0.01	< 0.001	(0.94, 0.00)		
					**Joint test for intervention effect**[c]	
Fixed effects	**Estimate**	**SE**	***p*-val**	**95% CI**	**Test statistic**	***p*-val**
Intervention arm main effect	0.07	0.12	0.13	(0.00, 2.27)	3.02	0.39
Intervention x time interaction	1.54	0.50	0.18	(0.81, 2.92)		
Intervention x time^2 interaction	0.98	0.01	0.23	(0.96, 1.01)		
Variance component	0.32	0.12		(0.15, 0.66)		

Abbreviation: GBP, General Best Practice; TBP, Tailored Best Practice; SE, Standard error; CI, Confidence interval
[a] Stratification factors used for randomization
[b] Agency name and identifiers suppressed to protect the confidentiality of health-related outcomes
[c] Joint Wald test was performed to assess whether there were significant differences in the trends between treatment groups

Supplementary Table S13J. Residents with ID/DD: Associations of GBP and TBP with group home-level COVID-19 incidence by time point

	Raw incidence rate[a]		Marginal mean incidence rate		Unadjusted	
Time period	**GBP**	**TBP**	**GBP**	**TBP**	**IRR**	**95% CI**
1-3 months[b]	0.00	0.00	0.08	0.02	0.21	(0.03, 1.41)
4-6 months	1.07	0.51	0.65	0.32	0.48	(0.21, 1.10)
7-9 months	1.84	1.54	2.31	1.95	0.81	(0.54, 1.21)
10-12 months	5.12	4.82	3.79	3.97	1.00	(0.71, 1.43)
13-15 months	3.05	2.94	2.87	2.74	0.91	(0.58, 1.43)

Abbreviation: GBP, General Best Practice; TBP, Tailored Best Practice; IRR, Incident rate ratio; CI, Confidence interval
[a] Raw and marginal mean COVID-19 incidence rate per 100 person-months
[b] Where month 1 is January, 2021.

Supplementary Table S14. Group home-level COVID-19 incidence rate ratios by time point among residents and staff, by race/ethnicity

Supplementary Table S14A. Treatment effect heterogeneity on COVID-19 incidence by race, residents

Fixed effects	Estimate	SE	*p*-val	95% CI	
Stratum 1[a]	Ref	-	-	-	
Stratum 2	0.80	0.19	0.36	(0.50, 1.29)	
Stratum 3	0.76	0.19	0.27	(0.47, 1.24)	
Stratum 4	0.58	0.16	0.05	(0.34, 1.00)	
Stratum 5	0.30	0.08	< 0.001	(0.17, 0.51)	
Stratum 6	0.55	0.13	0.02	(0.34, 0.89)	
Stratum 7	0.43	0.11	0.001	(0.27, 0.71)	
Stratum 8	0.43	0.11	0.001	(0.26, 0.70)	
Agency 1[b]	0.46	0.10	< 0.001	(0.30, 0.70)	
Agency 2	0.64	0.12	0.02	(0.45, 0.92)	
Agency 3	0.49	0.13	0.01	(0.29, 0.83)	
Agency 4	1.26	0.25	0.25	(0.85, 1.86)	
Agency 5	0.94	0.23	0.80	(0.58, 1.52)	
Agency 6	Ref	-	-	-	
Baseline home-level incidence	1.00	0.01	0.79	(0.97, 1.02)	
Race - White	Ref	-	-	-	
Race - Non-White	0.99	0.43	0.98	(0.42, 2.32)	
Race - Missing	1.81	1.39	0.44	(0.40, 8.15)	
Linear time trend	1.17	0.02	< 0.001	(1.12, 1.21)	
Intervention arm main effect	0.67	0.25	0.29	(0.32, 1.40)	
Intervention x time interaction	0.55	0.13	0.02	(0.34, 0.89)	
Race - White x time	Ref	-	-	-	
Race - Non-White x time	0.99	0.04	0.76	(0.92, 1.06)	

Race - Missing x time	0.92	0.06	0.22	(0.80, 1.05)		
					**Joint test for intervention effect[c]**	
Fixed effects	**Estimate**	**SE**	***p*-val**	**95% CI**	**Test statistic**	***p*-val**
Race - White x arm	Ref	-	-	-	2.19	0.701
Race - Non-White x arm	1.00	0.61	> 0.99	(0.30, 3.33)		
Race - Missing x arm	0.24	0.35	0.33	(0.01, 4.25)		
Race - White x arm x time	Ref	-	-	-		
Race - Non-White x arm x time	1.01	0.05	0.84	(0.92, 1.11)		
Race - Missing x arm x time	1.16	0.14	0.22	(0.92, 1.46)		
Variance component	0.58	0.11		(0.40, 0.85)		

Supplementary Table S14B. Group home-level COVID-19 incidence rate ratios by time point among residents, by race/ethnicity

	Raw incidence rate		Marginal mean incidence rate		Unadjusted	
Time period	GBP	TBP	GBP	TBP	IRR	95% CI
Non-Hispanic White						
1-3 months	0.00	0.00	0.32	0.44	0.75	(0.42, 1.34)
4-6 months	0.65	0.79	0.58	0.69	0.85	(0.55, 1.30)
7-9 months	0.93	1.76	1.03	1.10	0.95	(0.69, 1.31)
10-12 months	4.41	4.02	1.83	1.75	1.06	(0.81, 1.41)
13-15 months	1.93	1.97	3.25	2.78	1.19	(0.86, 1.67)
Non-White						
1-3 months	0.00	0.00	0.29	0.39	0.75	(0.31, 1.84)
4-6 months	0.23	0.43	0.52	0.61	0.84	(0.36, 1.96)
7-9 months	1.68	1.71	0.92	0.93	0.94	(0.41, 2.19)
10-12 months	4.05	3.00	1.64	1.43	1.06	(0.44, 2.55)
13-15 months	2.13	1.35	2.90	2.18	1.18	(0.45, 3.09)
Missing						
1-3 months	0.00	0.00	0.12	0.45	0.21	(0.02, 2.29)
4-6 months	0.00	0.56	0.25	0.55	0.29	(0.04, 2.13)
7-9 months	1.21	1.86	0.54	0.67	0.39	(0.07, 2.20)
10-12 months	2.06	2.98	1.14	0.82	0.53	(0.10, 2.68)
13-15 months	1.98	0.62	2.46	1.02	0.71	(0.13, 3.93)

Abbreviation: GBP, General Best Practice; TBP, Tailored Best Practice; IRR, Incident rate ratio; CI, Confidence interval
[a] Raw and marginal mean COVID-19 incidence rate per 100 person-months
[b] Where month 1 is January, 2021.

Supplementary Table S14C. Treatment effect heterogeneity on COVID-19 incidence by race, staff

Fixed effects	Estimate	SE	*p*-val	95% CI	
Stratum 1[a]	Ref	-	-	-	
Stratum 2	1.05	0.09	0.59	(0.88, 1.25)	
Stratum 3	0.89	0.09	0.22	(0.73, 1.07)	
Stratum 4	1.06	0.10	0.57	(0.87, 1.28)	
Stratum 5	1.03	0.10	0.75	(0.85, 1.25)	
Stratum 6	0.95	0.09	0.57	(0.78, 1.14)	
Stratum 7	0.82	0.08	0.05	(0.67, 1.00)	
Stratum 8	0.93	0.09	0.46	(0.76, 1.13)	
Agency 1[b]	0.76	0.05	< 0.001	(0.66, 0.87)	
Agency 2	0.47	0.04	< 0.001	(0.40, 0.56)	
Agency 3	1.10	0.09	0.22	(0.95, 1.29)	
Agency 4	1.23	0.11	0.02	(1.03, 1.47)	
Agency 5	0.93	0.08	0.39	(0.78, 1.10)	
Agency 6	Ref	-	-	-	
Baseline home-level incidence	1.01	0.01	0.17	(1.00, 1.03)	
Race - White	Ref	-	-	-	
Race - Non-White	0.99	0.27	0.97	(0.58, 1.68)	
Race - Missing	1.54	0.83	0.42	(0.54, 4.41)	
Linear time trend	1.17	0.02	< 0.001	(1.13, 1.22)	
Intervention arm main effect	1.12	0.39	0.74	(0.57, 2.22)	
Intervention x time interaction	0.95	0.09	0.57	(0.78, 1.14)	
Race - White x time	Ref	-	-	-	
Race - Non-White x time	0.99	0.02	0.52	(0.94, 1.03)	
Race - Missing x time	0.97	0.04	0.56	(0.89, 1.06)	
					**Joint test for intervention effect**[c]

Fixed effects	**Estimate**	**SE**	***p*-val**	**95% CI**	**Test statistic**	***p*-val**
Race - White x arm	Ref	-	-	-	4.07	0.40
Race - Non-White x arm	0.77	0.30	0.50	(0.36, 1.65)		
Race - Missing x arm	2.51	1.88	0.22	(0.58, 10.88)		
Race - White x arm x time	Ref	-	-	-		
Race - Non-White x arm x time	1.02	0.03	0.63	(0.95, 1.08)		
Race - Missing x arm x time	0.90	0.06	0.13	(0.79, 1.03)		
Variance component	0.00	0.00		(0.00, 0.00)		

Supplementary Table S14D. Group home-level COVID-19 incidence rate ratios by time point among staff, by race/ethnicity

	Raw incidence rate		Marginal mean incidence rate			Unadjusted
Time period	GBP	TBP	GBP	TBP	IRR	95% CI
Non-Hispanic White						
1-3 months	0.08	0.39	0.98	0.89	1.10	(0.65, 1.86)
4-6 months	1.49	0.89	1.57	1.45	1.08	(0.74, 1.57)
7-9 months	2.09	2.42	2.51	2.37	1.06	(0.82, 1.37)
10-12 months	7.82	6.40	3.91	3.76	1.04	(0.83, 1.30)
13-15 months	3.42	3.94	6.26	6.13	1.02	(0.75, 1.38)
Non-White						
1-3 months	0.11	0.20	0.76	0.86	0.85	(0.62, 1.17)
4-6 months	0.59	0.56	1.21	1.33	0.84	(0.59, 1.19)
7-9 months	2.70	3.03	1.94	2.06	0.83	(0.54, 1.27)
10-12 months	6.86	7.12	3.10	3.20	0.81	(0.48, 1.39)
13-15 months	2.17	2.05	4.93	4.96	0.80	(0.42, 1.53)
Missing						
1-3 months	1.00	1.48	2.89	1.96	1.88	(0.59, 5.99)
4-6 months	2.19	0.00	3.10	2.90	1.26	(0.43, 3.70)
7-9 months	5.12	3.32	3.34	4.30	0.84	(0.29, 2.46)
10-12 months	3.86	8.18	2.63	1.29	0.56	(0.18, 1.76)
13-15 months	1.27	3.57	3.68	6.54	0.38	(0.11, 1.34)

Abbreviation: GBP, General Best Practice; TBP, Tailored Best Practice; IRR, Incident rate ratio; CI, Confidence interval
[a] Raw and marginal mean COVID-19 incidence rate per 100 person-months
[b] Where month 1 is January, 2021.

Supplementary Table S15. Group home-level incidence rate ratios by time point among residents and staff of SMI homes, by race/ethnicity

There were also no significant differences in COVID-19 infection rates between arms by race when analyzed for residents with SMI; numbers became too small to analyze for residents with ID/DD alone.

Supplementary Table S15A. Treatment effect heterogeneity on COVID-19 incidence by race, SMI residents

Fixed effects	Estimate	SE	*p*-val	95% CI	
Stratum 5	Ref	-	-	-	
Stratum 6	1.70	0.46	0.05	(1.00, 2.88)	
Stratum 7	2.01	0.60	0.02	(1.12, 3.60)	
Stratum 8	1.54	0.44	0.13	(0.88, 2.71)	
Agency 1[b]	0.79	0.25	0.45	(0.43, 1.45)	
Agency 2	1.05	0.27	0.86	(0.63, 1.74)	
Agency 3	0.93	0.34	0.85	(0.45, 1.92)	
Agency 4	0.89	0.26	0.68	(0.50, 1.58)	
Agency 5	0.76	0.31	0.49	(0.34, 1.68)	
Agency 6	Ref	-	-	-	
Baseline home-level incidence	0.96	0.03	0.24	(0.90, 1.03)	
Race - White	Ref	-	-	-	
Race - Non-White	1.12	0.65	0.85	(0.36, 3.50)	
Race - Missing	2.26	2.14	0.39	(0.35, 14.47)	
Linear time trend	1.15	0.04	< 0.001	(1.09, 1.23)	
Intervention arm main effect	0.63	0.35	0.41	(0.21, 1.89)	
Intervention x time interaction	1.07	0.05	0.14	(0.98, 1.16)	
Race - White x time	Ref	-	-	-	

Race - Non-White x time	0.98	0.05	0.62	(0.89, 1.07)		
Race - Missing x time	0.88	0.08	0.16	(0.74, 1.05)		
					**Joint test for intervention effect**[c]	
Fixed effects	**Estimate**	**SE**	***p*-val**	**95% CI**	**Test statistic**	***p*-val**
Race - White x arm	Ref	-	-	-	1.06	0.90
Race - Non-White x arm	1.11	0.92	0.90	(0.22, 5.60)		
Race - Missing x arm	0.47	0.74	0.63	(0.02, 10.21)		
Race - White x arm x time	Ref	-	-	-		
Race - Non-White x arm x time	1.00	0.07	0.98	(0.88, 1.14)		
Race - Missing x arm x time	1.11	0.15	0.45	(0.85, 1.44)		
Variance component	0.68	0.17		(0.42, 1.11)		

Supplementary Table S15B. Associations of GBP and TBP with group home-level COVID-19 incidence by time point among SMI residents, by race/ethnicity

	**Raw incidence rate**		**Marginal mean incidence rate**			**Unadjusted**
Time period	**GBP**	**TBP**	**GBP**	**TBP**	**IRR**	**95% CI**
Non-Hispanic White						
1-3 months	0.00	0.00	0.21	0.31	0.76	(0.32, 1.82)
4-6 months	0.60	0.38	0.40	0.47	0.93	(0.48, 1.79)
7-9 months	0.95	1.54	0.74	0.73	1.14	(0.70, 1.85)
10-12 months	2.94	2.84	1.39	1.13	1.38	(0.90, 2.12)
13-15 months	1.66	0.97	2.60	1.73	1.69	(1.01, 2.80)
Non-White						
1-3 months	0.00	0.00	0.24	0.32	0.78	(0.25, 2.42)
4-6 months	0.40	0.15	0.42	0.46	0.88	(0.30, 2.61)
7-9 months	0.84	1.83	0.73	0.66	1.00	(0.33, 3.01)
10-12 months	4.53	2.08	1.26	0.94	1.13	(0.34, 3.69)
13-15 months	1.63	0.71	2.19	1.34	1.27	(0.34, 4.76)
Missing						
1-3 months	0.00	0.00	0.21	0.46	0.34	(0.03, 4.08)
4-6 months	0.00	0.79	0.37	0.49	0.38	(0.04, 3.41)

7-9 months	1.79	0.99	0.64	0.52	0.44	(0.06, 3.24)
10-12 months	2.98	2.17	1.12	0.54	0.49	(0.07, 3.61)
13-15 months	1.10	0.00	1.95	0.57	0.56	(0.07, 4.76)

Abbreviation: GBP, General Best Practice; TBP, Tailored Best Practice; IRR, Incident rate ratio; CI, Confidence interval
[a] Raw and marginal mean COVID-19 incidence rate per 100 person-months
[b] Where month 1 is January, 2021.

Supplementary Table S16. Treatment effect heterogeneity on COVID-19 incidence by agency and time point among residents and staff

There was, however, significant heterogeneity by agencies in differences of mean home-level COVID-19 incidence rates for residents between arms over time ($p < 0.001$). This was largely driven by trends in one agency, where GH-incidence of COVID-19 infections increased at a faster rate for TBP over months 4-9 relative to GBP, but subsequently also decreased at a faster rate at later time points. There were no statistically significant differences among residents when agencies were further stratified by GH type (i.e., SMI and ID/DD), although sample sizes became small.

Supplementary Table S16A. Resident: Treatment effect heterogeneity on COVID-19 incidence by agency

Fixed effects	Estimate	SE	*p*-val	95% CI	
Stratum 1[a]	Ref	-	-	-	
Stratum 2	0.79	0.19	0.32	(0.49, 1.26)	
Stratum 3	0.77	0.19	0.29	(0.48, 1.25)	
Stratum 4	0.58	0.16	0.04	(0.34, 0.98)	
Stratum 5	0.28	0.08	< 0.001	(0.16, 0.48)	
Stratum 6	0.53	0.13	0.008	(0.33, 0.85)	
Stratum 7	0.43	0.10	< 0.001	(0.26, 0.69)	
Stratum 8	0.43	0.11	< 0.001	(0.26, 0.70)	
Agency 1[b]	0.35	0.27	0.18	(0.08, 1.59)	
Agency 2	0.55	0.34	0.33	(0.17, 1.82)	
Agency 3	1.81	1.21	0.37	(0.49, 6.67)	
Agency 4	1.67	0.97	0.38	(0.53, 1.23)	
Agency 5	0.35	0.30	0.23	(0.06, 1.92)	
Agency 6	Ref	-	-	-	

Baseline home-level incidence	1.00	0.01	0.79	(0.97, 1.02)		
Linear time trend	1.17	0.03	< 0.001	(1.11, 1.23)		
Intervention arm main effect	0.53	0.25	0.17	(0.21, 1.32)		
Intervention x time interaction	1.05	0.04	0.19	(0.98, 1.13)		
Agency 1 x time	1.03	0.06	0.59	(0.92, 1.16)		
Agency 2 x time	0.97	0.05	0.53	(0.88, 1.07)		
Agency 3 x time	0.90	0.05	0.08	(0.81, 1.01)		
Agency 4 x time	0.95	0.04	0.30	(0.87, 1.04)		
Agency 5 x time	1.08	0.07	0.23	(0.95, 1.23)		
Agency 6 x time	Ref	-	-	-		
					**Joint test for intervention effect[c]**	
Fixed effects	**Estimate**	**SE**	***p*-val**	**95% CI**	**Test statistic**	***p*-val**
Agency 1 x arm	0.44	0.57	0.53	(0.03, 5.60)	273.24	< 0.001
Agency 2 x arm	3.05	2.47	0.17	(0.62, 14.95)		
Agency 3 x arm	0.26	0.34	0.31	(0.02, 3.47)		
Agency 4 x arm	0.48	0.41	0.39	(0.09, 2.59)		
Agency 5 x arm	19.05	21.52	0.009	(2.08, 174.40)		
Agency 6 x arm	Ref	-	-	-		
Agency 1 x arm x time	1.04	0.10	0.66	(0.86, 1.27)		
Agency 2 x arm x time	0.98	0.06	0.77	(0.87, 1.11)		
Agency 3 x arm x time	1.07	0.11	0.55	(0.86, 1.31)		
Agency 4 x arm x time	1.11	0.07	0.13	(0.97, 1.26)		
Agency 5 x arm x time	0.77	0.07	0.005	(0.65, 0.92)		
Agency 6 x arm x time	Ref	-	-	-		
Variance component	0.57	0.11		(0.39, 0.82)		

Supplementary Table S16B. Association of GBP and TBP with group home-level COVID-19 incidence by time point among residents, by agency

	Raw incidence rate		Marginal mean incidence rate			Unadjusted
Time period	GBP	TBP	GBP	TBP	IRR	95% CI
Agency 1						
1-3 months	0.00	0.00	0.07	0.20	0.34	(0.04, 2.65)
4-6 months	0.00	0.16	0.16	0.36	0.49	(0.08, 2.98)
7-9 months	0.00	1.07	0.37	0.63	0.71	(0.14, 3.67)
10-12 months	2.14	3.19	0.86	1.11	1.03	(0.21, 5.07)
13-15 months	1.17	1.11	1.99	1.96	1.50	(0.28, 7.94)
Agency 2						
1-3 months	0.00	0.00	0.38	0.31	1.60	(0.48, 5.39)
4-6 months	0.18	0.32	0.61	0.45	1.59	(0.50, 5.12)
7-9 months	2.72	0.95	0.97	0.65	1.58	(0.49, 5.10)
10-12 months	2.41	0.98	1.53	0.95	1.57	(0.47, 5.32)
13-15 months	2.28	1.27	2.43	1.38	1.56	(0.42, 5.79)
Agency 3						
1-3 months	0.00	0.00	0.18	0.69	0.14	(0.02, 1.09)
4-6 months	0.00	0.62	0.29	0.82	0.14	(0.03, 0.83)
7-9 months	0.26	2.72	0.48	0.96	0.15	(0.03, 0.71)
10-12 months	2.29	2.98	0.79	1.14	0.15	(0.03, 0.72)
13-15 months	0.00	0.00	1.31	1.34	0.16	(0.03, 0.86)
Agency 4						
1-3 months	0.00	0.00	0.28	0.88	0.34	(0.09, 1.22)
4-6 months	0.43	3.50	0.61	1.21	0.46	(0.14, 1.49)
7-9 months	0.79	0.36	1.32	1.69	0.62	(0.20, 1.94)
10-12 months	7.24	3.33	2.86	2.33	0.84	(0.26, 2.69)
13-15 months	5.15	3.77	6.16	3.22	1.13	(0.32, 3.96)
Agency 5						
1-3 months	0.00	0.00	1.43	0.26	6.77	(1.05, 43.82)
4-6 months	2.37	0.07	1.54	0.53	4.55	(0.77, 27.07)
7-9 months	3.26	0.94	1.65	1.07	3.06	(0.52, 18.00)
10-12 months	2.22	5.53	1.78	2.16	2.06	(0.33, 12.91)
13-15 months	1.28	4.99	1.91	4.36	1.39	(0.19, 9.91)
Agency 6						

1-3 months	0.00	0.00	0.33	0.52	0.61	(0.29, 1.26)
4-6 months	0.34	0.24	0.61	0.84	0.70	(0.40, 1.22)
7-9 months	1.14	2.19	1.12	1.34	0.81	(0.53, 1.23)
10-12 months	5.46	5.91	2.07	2.13	0.94	(0.65, 1.35)
13-15 months	2.13	1.76	3.80	3.40	1.08	(0.71, 1.65)

Abbreviation: GBP, General Best Practice; TBP, Tailored Best Practice; IRR, Incident rate ratio; CI, Confidence interval
[a] Raw and marginal mean COVID-19 incidence rate per 100 person-months
[b] Where month 1 is January, 2021.

Supplementary Table S16C. Staff: Treatment effect heterogeneity on COVID-19 incidence by agency

Fixed effects	Estimate	SE	*p*-val	95% CI	
Stratum 1[a]	Ref	-	-	-	
Stratum 2	1.03	0.09	0.71	(0.87, 1.23)	
Stratum 3	0.92	0.09	0.38	(0.76, 1.11)	
Stratum 4	1.07	0.10	0.46	(0.89, 1.29)	
Stratum 5	1.00	0.10	0.99	(0.83, 1.21)	
Stratum 6	0.91	0.09	0.33	(0.76, 1.10)	
Stratum 7	0.88	0.09	0.18	(0.72, 1.06)	
Stratum 8	0.99	0.09	0.88	(0.82, 1.19)	
Agency 1[b]	0.49	0.15	0.02	(0.27, 0.90)	
Agency 2	0.46	0.16	0.02	(0.24, 0.90)	
Agency 3	1.17	0.34	0.59	(0.66, 2.06)	
Agency 4	0.82	0.32	0.61	(0.37, 1.18)	
Agency 5	0.80	0.29	0.53	(0.39, 1.62)	
Agency 6	Ref	-	-	-	
Baseline home-level incidence	1.01	0.01	0.22	(0.99, 1.03)	
Linear time trend	1.15	0.02	< 0.001	(1.12, 1.18)	
Intervention arm main effect	0.96	0.23	0.87	(0.61, 1.52)	
Intervention x time interaction	1.00	0.02	0.84	(0.97, 1.04)	
Agency 1 x time	1.05	0.03	0.08	(0.99, 1.10)	
Agency 2 x time	1.02	0.03	0.52	(0.96, 1.07)	
Agency 3 x time	1.00	0.02	> 0.99	(0.95, 1.05)	
Agency 4 x time	1.02	0.03	0.50	(0.96, 1.09)	
Agency 5 x time	1.01	0.03	0.66	(0.96, 1.08)	
Agency 6 x time	Ref	-	-	-	
					**Joint test for intervention effect[c]**

Fixed effects	Estimate	SE	*p*-val	95% CI	Test statistic	*p*-val
Agency 1 x arm	0.64	0.30	0.34	(0.26, 1.59)	13.00	0.22
Agency 2 x arm	1.06	0.52	0.91	(0.40, 2.77)		
Agency 3 x arm	1.22	0.52	0.65	(0.52, 2.82)		
Agency 4 x arm	0.95	0.49	0.92	(0.34, 2.64)		
Agency 5 x arm	1.46	0.74	0.46	(0.54, 3.96)		
Agency 6 x arm	Ref	-	-	-		
Agency 1 x arm x time	1.02	0.04	0.55	(0.95, 1.10)		
Agency 2 x arm x time	0.97	0.04	0.46	(0.89, 1.05)		
Agency 3 x arm x time	0.98	0.04	0.52	(0.91, 1.05)		
Agency 4 x arm x time	1.03	0.04	0.51	(0.95, 1.12)		
Agency 5 x arm x time	0.97	0.04	0.46	(0.89, 1.05)		
Agency 6 x arm x time	Ref	-	-	-		
Variance component	0.00	0.00		(0.00, 0.00)		

Supplementary Table S16D. Association of GBP and TBP with group home-level COVID-19 incidence by time point among staff, by agency

	Raw incidence rate		Marginal mean incidence rate			Unadjusted
Time period	GBP	TBP	GBP	TBP	IRR	95% CI
Agency 1						
1-3 months	0.00	0.00	0.40	0.60	0.77	(0.39, 1.52)
4-6 months	0.35	0.80	0.75	1.03	0.95	(0.51, 1.77)
7-9 months	1.73	1.36	1.40	1.78	1.17	(0.64, 2.15)
10-12 months	5.42	6.71	2.61	3.06	1.45	(0.77, 2.72)
13-15 months	2.84	3.66	4.86	5.26	1.79	(0.90, 3.57)
Agency 2						
1-3 months	0.05	0.05	0.49	0.52	0.99	(0.47, 2.10)
4-6 months	0.21	0.21	0.71	0.83	0.97	(0.49, 1.92)
7-9 months	2.35	2.25	1.05	1.32	0.94	(0.48, 1.83)
10-12 months	1.95	4.02	1.54	2.10	0.92	(0.46, 1.83)
13-15 months	1.31	1.83	2.26	3.34	0.89	(0.42, 1.91)
Agency 3						
1-3 months	0.46	0.31	1.39	1.24	1.10	(0.59, 2.06)
4-6 months	1.09	1.12	1.98	1.86	1.04	(0.59, 1.85)
7-9 months	3.90	3.39	2.82	2.81	0.98	(0.56, 1.73)
10-12 months	10.91	11.40	4.00	4.23	0.93	(0.51, 1.69)
13-15 months	1.73	2.46	5.68	6.37	0.87	(0.44, 1.72)
Agency 4						
1-3 months	0.10	0.36	0.86	0.85	1.08	(0.47, 2.49)
4-6 months	0.37	1.10	1.53	1.37	1.27	(0.58, 2.79)
7-9 months	2.17	2.44	2.71	2.21	1.50	(0.69, 3.24)
10-12 months	12.16	6.54	4.81	3.56	1.76	(0.80, 3.89)
13-15 months	3.12	4.85	8.54	5.74	2.08	(0.89, 4.82)
Agency 5						
1-3 months	0.16	0.73	1.11	0.85	1.35	(0.61, 2.98)
4-6 months	1.91	0.37	1.60	1.33	1.29	(0.62, 2.69)
7-9 months	2.76	2.85	2.30	2.09	1.24	(0.61, 2.53)
10-12 months	5.39	6.03	3.32	3.27	1.19	(0.57, 2.49)
13-15 months	3.23	2.78	4.80	5.12	1.14	(0.51, 2.54)
Agency 6						

1-3 months	0.19	0.35	1.04	1.06	0.97	(0.69, 1.38)
4-6 months	0.82	0.56	1.59	1.61	0.99	(0.77, 1.27)
7-9 months	3.72	3.93	2.42	2.42	1.00	(0.84, 1.19)
10-12 months	7.38	7.73	3.70	3.65	1.01	(0.87, 1.18)
13-15 months	2.47	2.42	5.63	5.49	1.02	(0.83, 1.26)

Abbreviation: GBP, General Best Practice; TBP, Tailored Best Practice; IRR, Incident rate ratio; CI, Confidence interval
[a] Raw and marginal mean COVID-19 incidence rate per 100 person-months
[b] Where month 1 is January, 2021.

Supplementary Table S16E. Residents with IDD: Treatment effect heterogeneity on COVID-19 incidence by agency

Fixed effects	**Estimate**	**SE**	***p*-val**	**95% CI**		
Stratum 1[a]	Ref	-	-	-		
Stratum 2	0.84	0.18	0.43	(0.55, 1.29)		
Stratum 3	0.73	0.17	0.18	(0.47, 1.15)		
Stratum 4	0.63	0.15	0.06	(0.39, 1.01)		
Agency 1[b]	0.21	0.26	0.20	(0.02, 2.31)		
Agency 2	0.66	0.63	0.66	(0.10, 4.26)		
Agency 3	0.70	0.85	0.77	(0.06, 7.66)		
Agency 4	2.73	1.96	0.16	(0.67, 1.28)		
Agency 5	0.42	0.49	0.46	(0.04, 4.15)		
Agency 6	Ref	-	-	-		
Baseline home-level incidence	1.00	0.01	0.96	(0.97, 1.02)		
Linear time trend	1.20	0.04	< 0.001	(1.12, 1.28)		
Intervention arm main effect	0.58	0.38	0.41	(0.16, 2.08)		
Intervention x time interaction	1.02	0.05	0.73	(0.92, 1.12)		
Agency 1 x time	1.03	0.10	0.74	(0.86, 1.24)		
Agency 2 x time	0.94	0.07	0.46	(0.81, 1.10)		
Agency 3 x time	0.91	0.10	0.37	(0.74, 1.12)		
Agency 4 x time	0.92	0.05	0.17	(0.83, 1.03)		
Agency 5 x time	1.08	0.09	0.38	(0.91, 1.28)		
Agency 6 x time	Ref	-	-	-		
					**Joint test for intervention effect**[c]	
Fixed effects	**Estimate**	**SE**	***p*-val**	**95% CI**	**Test statistic**	***p*-val**
Agency 1 x arm	0.26	0.62	0.57	(0.00, 25.88)	12.62	0.25
Agency 2 x arm	0.62	0.88	0.74	(0.04, 9.89)		
Agency 3 x arm	0.67	1.28	0.84	(0.02, 27.89)		

Agency 4 x arm	0.62	0.67	0.66	(0.07, 5.15)	
Agency 5 x arm	16.10	23.38	0.06	(0.93, 277.34)	
Agency 6 x arm	Ref	-	-	-	
Agency 1 x arm x time	1.10	0.19	0.60	(0.78, 1.55)	
Agency 2 x arm x time	1.10	0.12	0.41	(0.88, 1.37)	
Agency 3 x arm x time	1.09	0.17	0.58	(0.80, 1.48)	
Agency 4 x arm x time	1.10	0.09	0.28	(0.93, 1.29)	
Agency 5 x arm x time	0.78	0.09	0.03	(0.62, 0.98)	
Agency 6 x arm x time	Ref	-	-	-	
Variance component	0.30	0.12		(0.14, 0.64)	

Supplementary Table S16F. Association of GBP and TBP with group home-level COVID-19 incidence by time point among ID/DD residents, by agency

	Raw incidence rate		Marginal mean incidence rate			Unadjusted
Time period	GBP	TBP	GBP	TBP	IRR	95% CI
Agency 1						
1-3 months	0.00	0.00	0.04	0.16	0.23	(0.01, 9.82)
4-6 months	0.00	0.00	0.10	0.30	0.36	(0.02, 8.42)
7-9 months	0.00	1.85	0.25	0.57	0.54	(0.04, 8.40)
10-12 months	1.59	1.66	0.66	1.09	0.83	(0.07, 10.54)
13-15 months	1.22	1.92	1.74	2.05	1.27	(0.09, 17.51)
Agency 2						
1-3 months	0.00	0.00	0.18	0.37	0.42	(0.05, 3.77)
4-6 months	0.00	0.73	0.35	0.54	0.49	(0.07, 3.63)
7-9 months	2.76	1.44	0.71	0.78	0.57	(0.08, 3.83)
10-12 months	0.00	0.00	1.42	1.12	0.66	(0.10, 4.50)
13-15 months	3.80	2.23	2.85	1.63	0.77	(0.10, 5.89)
Agency 3						
1-3 months	0.00	0.00	0.20	0.39	0.40	(0.02, 8.77)
4-6 months	0.00	0.00	0.35	0.51	0.41	(0.03, 6.54)
7-9 months	0.00	2.26	0.62	0.66	0.42	(0.03, 5.57)
10-12 months	3.51	2.16	1.10	0.85	0.44	(0.03, 5.57)
13-15 months	0.00	0.00	1.95	1.11	0.45	(0.03, 6.59)
Agency 4						
1-3 months	0.00	0.00	0.68	1.50	0.40	(0.09, 1.78)
4-6 months	0.95	5.64	1.28	2.04	0.43	(0.11, 1.74)
7-9 months	1.16	0.37	2.43	2.78	0.48	(0.12, 1.84)
10-12 months	11.90	4.70	4.59	3.79	0.52	(0.13, 2.14)
13-15 months	6.30	5.96	8.67	5.16	0.57	(0.12, 2.69)
Agency 5						
1-3 months	0.00	0.00	1.85	0.35	5.94	(0.54, 64.97)
4-6 months	3.68	0.00	2.02	0.76	3.77	(0.37, 38.02)
7-9 months	2.58	1.04	2.21	1.65	2.39	(0.24, 24.10)
10-12 months	3.45	10.59	2.42	3.58	1.52	(0.14, 16.54)
13-15 months	1.99	5.24	2.65	7.79	0.97	(0.08, 12.22)
Agency 6						

1-3 months	0.00	0.00	0.47	0.68	0.61	(0.22, 1.67)
4-6 months	0.18	0.38	0.85	1.17	0.65	(0.31, 1.36)
7-9 months	1.79	2.70	1.55	2.02	0.68	(0.40, 1.16)
10-12 months	6.68	9.13	2.81	3.48	0.72	(0.46, 1.12)
13-15 months	2.79	3.20	5.10	6.00	0.76	(0.44, 1.29)

Abbreviation: GBP, General Best Practice; TBP, Tailored Best Practice; IRR, Incident rate ratio; CI, Confidence interval
[a] Raw and marginal mean COVID-19 incidence rate per 100 person-months
[b] Where month 1 is January, 2021.

Supplementary Table S16G. Residents with SMI: Treatment effect heterogeneity on COVID-19 incidence by agency

Fixed effects	Estimate	SE	*p*-val	95% CI		
Stratum 5	Ref	-	-	-		
Stratum 6	1.68	0.44	0.05	(1.00, 2.81)		
Stratum 7	2.11	0.61	0.01	(1.20, 3.71)		
Stratum 8	1.64	0.46	0.08	(0.94, 2.85)		
Agency 1[b]	0.54	0.54	0.54	(0.08, 3.86)		
Agency 2	0.59	0.47	0.51	(0.12, 2.82)		
Agency 3	3.69	3.05	0.12	(0.73, 18.69)		
Agency 4	0.71	0.72	0.73	(0.10, 1.21)		
Agency 5	0.26	0.33	0.29	(0.02, 3.16)		
Agency 6	Ref	-	-	-		
Baseline home-level incidence	0.95	0.04	0.23	(0.89, 1.03)		
Linear time trend	1.13	0.04	< 0.001	(1.05, 1.21)		
Intervention arm main effect	0.47	0.31	0.26	(0.13, 1.73)		
Intervention x time interaction	1.09	0.06	0.10	(0.98, 1.20)		
Agency 1 x time	1.05	0.08	0.51	(0.90, 1.23)		
Agency 2 x time	1.00	0.06	0.95	(0.89, 1.14)		
Agency 3 x time	0.92	0.06	0.25	(0.81, 1.06)		
Agency 4 x time	0.99	0.08	0.92	(0.84, 1.17)		
Agency 5 x time	1.09	0.10	0.39	(0.90, 1.31)		
Agency 6 x time	Ref	-	-	-		
					**Joint test for intervention effect**[c]	
Fixed effects	**Estimate**	**SE**	***p*-val**	**95% CI**	**Test statistic**	***p*-val**
Agency 1 x arm	0.64	1.02	0.78	(0.03, 14.61)	17.52	0.06
Agency 2 x arm	6.17	6.41	0.08	(0.81, 47.26)		
Agency 3 x arm	0.19	0.39	0.42	(0.00, 9.98)		

Agency 4 x arm	0.36	0.53	0.48	(0.02, 6.27)	
Agency 5 x arm	24.98	46.45	0.08	(0.65, 955.70)	
Agency 6 x arm	Ref	-	-	-	
Agency 1 x arm x time	1.00	0.12	0.98	(0.79, 1.27)	
Agency 2 x arm x time	0.92	0.08	0.31	(0.78, 1.08)	
Agency 3 x arm x time	0.99	0.17	0.95	(0.71, 1.37)	
Agency 4 x arm x time	1.13	0.13	0.29	(0.90, 1.41)	
Agency 5 x arm x time	0.75	0.12	0.07	(0.55, 1.03)	
Agency 6 x arm x time	Ref	-	-	-	
Variance component	0.60	0.15		(0.36, 0.99)	

Supplementary Table S16H. Association of GBP and TBP with group home-level COVID-19 incidence by time point among SMI residents, by agency

	Raw incidence rate		Marginal mean incidence rate			Unadjusted
Time period	GBP	TBP	GBP	TBP	IRR	95% CI
Agency 1						
1-3 months	0.00	0.00	0.10	0.23	0.46	(0.04, 5.62)
4-6 months	0.00	0.35	0.21	0.39	0.70	(0.07, 6.55)
7-9 months	0.00	0.11	0.46	0.66	1.06	(0.13, 8.53)
10-12 months	2.79	5.04	1.01	1.11	1.61	(0.20, 12.72)
13-15 months	1.11	0.13	2.22	1.88	2.44	(0.27, 21.83)
Agency 2						
1-3 months	0.00	0.00	0.47	0.28	2.97	(0.67, 13.19)
4-6 months	0.33	0.00	0.69	0.40	3.01	(0.69, 13.05)
7-9 months	2.68	0.57	1.01	0.59	3.05	(0.68, 13.74)
10-12 months	4.50	1.73	1.49	0.86	3.10	(0.63, 15.30)
13-15 months	0.97	0.53	2.19	1.27	3.14	(0.55, 17.88)
Agency 3						
1-3 months	0.00	0.00	0.21	0.90	0.09	(0.00, 1.85)
4-6 months	0.00	1.38	0.30	1.02	0.09	(0.01, 1.02)
7-9 months	0.53	3.29	0.43	1.17	0.09	(0.01, 0.72)
10-12 months	1.08	3.99	0.61	1.33	0.08	(0.01, 0.71)
13-15 months	0.00	0.00	0.86	1.51	0.08	(0.01, 1.00)
Agency 4						
1-3 months	0.00	0.00	0.08	0.37	0.31	(0.03, 3.08)
4-6 months	0.00	1.03	0.22	0.52	0.55	(0.07, 4.65)
7-9 months	0.49	0.35	0.57	0.75	0.99	(0.13, 7.63)
10-12 months	3.41	1.63	1.49	1.06	1.77	(0.23, 13.79)
13-15 months	4.19	1.04	3.88	1.50	3.19	(0.37, 27.38)
Agency 5						
1-3 months	0.00	0.00	0.95	0.17	8.36	(0.40, 172.50)
4-6 months	0.00	0.15	0.97	0.31	5.90	(0.36, 96.34)
7-9 months	4.49	0.85	0.99	0.58	4.16	(0.27, 64.60)
10-12 months	0.00	0.47	1.01	1.07	2.93	(0.16, 52.53)
13-15 months	0.00	4.75	1.03	1.99	2.07	(0.08, 50.47)
Agency 6						

1-3 months	0.00	0.00	0.22	0.40	0.61	(0.22, 1.70)
4-6 months	0.50	0.11	0.40	0.57	0.78	(0.36, 1.72)
7-9 months	0.54	1.71	0.75	0.83	1.01	(0.55, 1.84)
10-12 months	4.34	2.93	1.39	1.20	1.30	(0.76, 2.22)
13-15 months	1.51	0.44	2.59	1.74	1.67	(0.89, 3.14)

Abbreviation: GBP, General Best Practice; TBP, Tailored Best Practice; IRR, Incident rate ratio; CI, Confidence interval
[a] Raw and marginal mean COVID-19 incidence rate per 100 person-months
[b] Where month 1 is January, 2021.

Supplementary Table S17. Sensitivity Analysis excluding Agency 5

To assess the impact of including data from this agency on the overall effect in the primary resident COVID-19 outcome, a sensitivity analysis was run excluding it from the model. We observed differences in trends after the exclusion (Bonferroni-adjusted $p < 0.001$). Differences were such that TBP was associated with reductions in mean COVID-19 incident rates in earlier periods (IRR: 0.07–0.28 over months 1-9) and increased rates at later periods (IRR: 1.29–1.58 over months 10-15).

Supplementary Table S17A. Resident (ID/DD and SMI combined) excluding Agency 5: Associations of GBP and TBP with group home-level COVID-19 incidence (n=385)

Fixed effects	**Estimate**	**SE**	***p*-val**	**95% CI**	
Stratum 2[a]	0.77	0.19	0.29	(0.47, 1.25)	
Stratum 3	0.75	0.20	0.27	(0.45, 1.25)	
Stratum 4	0.54	0.16	0.03	(0.31, 0.96)	
Stratum 5	0.29	0.08	< 0.001	(0.17, 0.50)	
Stratum 6	0.57	0.14	0.03	(0.35, 0.93)	
Stratum 7	0.44	0.11	0.002	(0.26, 0.73)	
Stratum 8	0.41	0.11	< 0.001	(0.24, 0.68)	
Agency 1	0.46	0.10	< 0.001	(0.31, 0.70)	
Agency 2	0.63	0.11	0.009	(0.44, 0.89)	
Agency 3	0.49	0.13	0.006	(0.29, 0.81)	
Agency 4	1.26	0.25	0.25	(0.85, 0.81)	
Agency 6	Ref	-	-	-	
Baseline home-level incidence	1.00	0.01	0.79	(0.97, 1.02)	
Linear time trend	4.18	0.69	< 0.001	(3.03, 5.78)	
Quadratic time trend (time^2)	0.94	0.01	< 0.001	(0.93, 0.95)	

					Joint test for intervention effect[b]	
Fixed effects	**Estimate**	**SE**	***p*-val**	**95% CI**	**Test statistic**	***p*-val***
Intervention arm main effect	0.01	0.02	0.007	(0.00, 0.31)	20.32	< 0.001
Intervention x time interaction	1.88	0.53	0.03	(1.08, 3.27)		
Intervention x time^2 interaction	0.98	0.01	0.09	(0.96, 1.00)		
Variance component	0.56	0.11		(0.38, 0.84)		

Abbreviation: GBP, General Best Practice; TBP, Tailored Best Practice; SE, Standard error; CI, Confidence interval
[a] Stratification factors used for randomization
[b] Joint Wald test was performed to assess whether there were significant differences in the trends between treatment groups
* p-value was adjusted for multiple comparisons using the Bonferroni correction.

Supplementary Table S17B. Resident (ID/DD and SMI combined) excluding Agency 5: Associations of GBP and TBP with group home-level COVID-19 incidence by time point (n=385)

	Raw incidence rate		Marginal mean incidence rate		Unadjusted	
Time period	**TBP**	**GBP**	**TBP**	**GBP**	**IRR**	**95% CI**
1-3 months	0.00	0.00	0.03	0.01	0.07	(0.01, 0.42)
4-6 months	0.71	0.39	0.41	0.19	0.28	(0.13, 0.60)
7-9 months	1.50	1.31	1.85	1.51	0.73	(0.53, 1.01)
10-12 months	3.91	4.20	2.86	3.32	1.29	(0.98, 1.71)
13-15 months	1.91	2.26	1.52	2.02	1.58	(1.08, 2.30)

Abbreviation: GBP, General Best Practice; TBP, Tailored Best Practice; IRR, Incident rate ratio; CI, Confidence interval
[a] Raw and marginal mean COVID-19 incidence rate per 100 person-months
[b] Where month 1 is January, 2021.

Supplementary Table S18. Association of GBP and TBP practices with home-level resident hospitalization rates between 1/2021-7/2022 (n=415)

Fixed effects	HR	SE	*p*-value	95% CI
Intervention effect	0.71	0.28	0.38	(0.33, 1.53)
Stratum 1	Ref	-	-	-
Stratum 2	0.53	0.53	0.53	(0.07, 3.77)
Stratum 3	0.98	1.00	0.98	(0.13, 7.31)
Stratum 4	1.71	1.50	0.54	(0.31, 9.52)
Stratum 5	0.50	0.46	0.46	(0.08, 3.04)
Stratum 6	0.79	0.68	0.78	(0.15, 4.23)
Stratum 7	0.46	0.47	0.44	(0.06, 3.34)
Stratum 8	1.37	1.10	0.70	(0.28, 6.60)
Agency 1	0.12	0.13	0.04	(0.02, 0.93)
Agency 2	0.52	0.25	0.18	(0.20, 1.34)
Agency 3	0.00	0.00	0.99	(0.00, 1.34)
Agency 4	0.17	0.18	0.10	(0.02, 1.40)
Agency 5	0.00	0.00	0.99	(0.00, 0.00)

Supplementary Table S19. Association of home-level characteristics with home-level COVID-19 infection incident rates

Among residents with ID/DD, home-level infection rates were inversely associated with prevalence of prior resident infection (IRR: 0.99 for GHs with a 1% higher baseline prevalence of prior infection, $p \leq 0.001$, 95% CI: 0.98–0.99). Among residents with SMI, home-level infection rates were inversely associated with baseline mean age of staff (IRR: 0.95, p=0.02, 95% CI: 0.91–0.99). Home-level staff infection rates were inversely associated with prevalence of prior resident infection (IRR: 0.998 for GHs with a 1% higher baseline prevalence of prior infection, p=0.02, 95% CI: 0.996-0.999) and prevalence of prior staff infection (IRR: 0.996 for GHs with a 1% higher baseline prevalence of prior infection, p=0.04, 95% CI: 0.991-0.999).

Supplementary Table S19A. Resident: Association of home-level characteristics with home-level COVID-19 infection incident rates (n=415)

Fixed effects	IRR	SE	p-value	95% CI
Stratum 1	Ref	-	-	-
Stratum 2	0.58	0.15	0.03	(0.35, 0.95)
Stratum 3	0.76	0.20	0.29	(0.46, 1.26)
Stratum 4	0.44	0.13	0.007	(0.24, 0.80)
Stratum 5	0.24	0.07	< 0.001	(0.14, 0.44)
Stratum 6	0.44	0.13	0.006	(0.25, 0.79)
Stratum 7	0.36	0.11	< 0.001	(0.20, 0.66)
Stratum 8	0.40	0.13	0.004	(0.21, 0.75)
Baseline home-level resident incidence	1.02	0.01	0.19	(0.99, 1.05)
Agency 1	0.43	0.10	< 0.001	(0.27, 0.69)
Agency 2	0.69	0.14	0.06	(0.47, 1.02)

Agency 3	0.40	0.12	0.002	(0.23, 0.71)
Agency 4	1.07	0.22	0.75	(0.71, 1.60)
Agency 5	0.86	0.23	0.56	(0.51, 1.44)
Agency 6	Ref	-	-	-
Bedrooms	1.00	0.02	0.90	(0.96, 1.04)
Home intensity	1.05	0.17	0.78	(0.76, 1.44)
Staff-to-resident ratio	1.08	0.06	0.16	(0.97, 1.20)
Prevalence of vaccinated residents	1.01	0.00	0.02	(1.00, 1.01)
Prevalence of vaccinated vax staff	1.00	0.00	0.73	(0.99, 1.01)
Prevalence of prior resident infection	0.99	0.00	< 0.001	(0.98, 0.99)
Prevalence of prior staff infection	1.00	0.00	0.39	(0.99, 1.01)
Mean age of resident	1.00	0.01	0.45	(0.98, 1.01)
Mean age of staff	0.99	0.01	0.37	(0.96, 1.02)
Proportion of non-white residents	1.00	0.00	0.71	(1.00, 1.01)
Proportion of non-white staff	1.00	0.00	0.27	(1.00, 1.01)
Proportion of male residents	1.00	0.00	0.54	(1.00, 1.01)
Proportion of male staff	0.99	0.00	0.13	(0.99, 1.00)
City-level incidence rate	1.03	0.09	0.70	(0.88, 1.22)
Linear time trend	4.01	0.63	< 0.001	(2.95, 5.45)
Quadratic time trend (time^2)	0.94	0.01	< 0.001	(0.93, 0.96)
Intervention arm main effect	0.08	0.11	0.07	(0.00, 1.20)
Intervention x time interaction	1.46	0.37	0.13	(0.89, 2.38)
Intervention x time^2 interaction	0.99	0.01	0.26	(0.97, 1.01)

Variance component	0.46	0.10	(0.30, 0.70)

*Staff to resident ratio is the number of unique staff who worked in the home during baseline divided by the number of resident beds in the home. Prevalence of vaccinated individuals and prior infection, mean age, proportion of non-white individuals and city-level incidence rate per 100 people were calculated at baseline.

Supplementary Table S19B. Residents with IDD: Association of home-level characteristics with home-level COVID-19 infection incident rates (n=206)

Fixed effects	IRR	SE	p-value	95% CI
Stratum 1	Ref	-	-	-
Stratum 2	0.58	0.14	0.02	(0.37, 0.93)
Stratum 3	0.68	0.17	0.13	(0.42, 1.11)
Stratum 4	0.43	0.13	0.004	(0.24, 0.76)
Baseline home-level resident incidence	1.02	0.01	0.13	(0.99, 1.05)
Agency 1	0.31	0.10	< 0.001	(0.17, 0.58)
Agency 2	0.46	0.13	0.006	(0.26, 0.80)
Agency 3	0.24	0.09	< 0.001	(0.11, 0.51)
Agency 4	1.20	0.38	0.57	(0.64, 2.24)
Agency 5	1.09	0.35	0.80	(0.58, 2.04)
Agency 6	Ref	-	-	-
Bedrooms	0.99	0.07	0.90	(0.87, 1.13)
Home intensity	0.93	0.19	0.73	(0.63, 1.38)
Staff-to-resident ratio	1.05	0.07	0.49	(0.92, 1.19)
Prevalence of vaccinated residents	1.00	0.00	0.46	(1.00, 1.01)
Prevalence of vaccinated vax staff	1.00	-0.38	0.70	(0.99, 1.01)
Prevalence of prior resident infection	0.99	0.00	< 0.001	(0.98, 1.00)
Prevalence of prior staff infection	0.99	0.01	0.24	(0.98, 1.00)
Mean age of resident	1.00	0.01	0.57	(0.98, 1.01)
Mean age of staff	1.01	0.02	0.59	(0.97, 1.05)
Proportion of non-white residents	1.00	0.00	0.81	(0.99, 1.01)

Proportion of non-white staff	1.00	0.00	0.82	(0.99, 1.01)
Proportion of male residents	1.00	0.00	0.67	(1.00, 1.01)
Proportion of male staff	1.00	0.00	0.62	(0.99, 1.01)
City-level incidence rate	1.12	0.13	0.34	(0.89, 1.41)
Linear time trend	2.92	0.57	< 0.001	(1.99, 4.26)
Quadratic time trend (time^2)	0.96	0.01	< 0.001	(0.94, 0.97)
Intervention arm main effect	0.07	0.13	0.14	(0.00, 2.40)
Intervention x time interaction	1.54	0.50	0.19	(0.81, 2.91)
Intervention x time^2 interaction	0.98	0.01	0.23	(0.96, 1.01)
Variance component	0.18	0.10		(0.06, 0.53)

*Staff to resident ratio is the number of unique staff who worked in the home during baseline divided by the number of resident beds in the home. Prevalence of vaccinated individuals and prior infection, mean age, proportion of non-white individuals and city-level incidence rate per 100 people were calculated at baseline.

Supplementary Table S19C. Residents with SMI: Association of home-level characteristics with home-level COVID-19 infection incident rates (n=209)

Fixed effects	IRR	SE	p-value	95% CI
Stratum 5	Ref	-	-	-
Stratum 6	1.53	0.43	0.13	(0.88, 2.65)
Stratum 7	1.90	0.64	0.06	(0.98, 3.68)
Stratum 8	1.47	0.50	0.26	(0.76, 2.86)
Baseline home-level resident incidence	0.97	0.04	0.47	(0.89, 1.06)
Agency 1	0.75	0.30	0.46	(0.34, 1.62)
Agency 2	1.25	0.39	0.48	(0.68, 2.32)
Agency 3	0.70	0.32	0.44	(0.28, 1.73)
Agency 4	0.77	0.25	0.42	(0.41, 1.44)
Agency 5	0.56	0.24	0.18	(0.24, 1.32)
Agency 6	Ref	-	-	-
Bedrooms	1.00	0.03	0.93	(0.95, 1.05)
Home intensity	1.24	0.38	0.48	(0.68, 2.24)
Staff-to-resident ratio	1.06	0.11	0.57	(0.87, 1.29)
Prevalence of vaccinated residents	1.01	0.00	0.14	(1.00, 1.01)
Prevalence of vaccinated vax staff	1.00	0.43	0.66	(0.99, 1.01)
Prevalence of prior resident infection	0.99	0.01	0.09	(0.97, 1.00)
Prevalence of prior staff infection	1.00	0.01	0.90	(0.99, 1.01)
Mean age of resident	1.00	0.01	0.70	(0.97, 1.02)
Mean age of staff	0.95	0.02	0.02	(0.91, 0.99)
Proportion of non-white residents	1.00	0.01	0.72	(0.99, 1.01)

Proportion of non-white staff	1.01	0.01	0.18	(1.00, 1.02)
Proportion of male residents	1.00	0.00	0.82	(0.99, 1.01)
Proportion of male staff	0.99	0.01	0.08	(0.98, 1.00)
City-level incidence rate	1.02	0.13	0.86	(0.80, 1.30)
Linear time trend	6.45	1.72	< 0.001	(3.82, 10.89)
Quadratic time trend (time^2)	0.92	0.01	< 0.001	(0.90, 0.94)
Intervention arm main effect	0.19	0.41	0.44	(0.00, 14.01)
Intervention x time interaction	1.18	0.46	0.67	(0.55, 2.55)
Intervention x time^2 interaction	1.00	0.02	0.98	(0.97, 1.03)
Variance component	0.54	0.15		(0.32, 0.93)

*Staff to resident ratio is the number of unique staff who worked in the home during baseline divided by the number of resident beds in the home. Prevalence of vaccinated individuals and prior infection, mean age, proportion of non-white individuals and city-level incidence rate per 100 people were calculated at baseline.

Supplementary Table S19D. Staff: Association of home-level characteristics with home-level COVID-19 infection incident rates (n=415)

Fixed effects	IRR	SE	p-value	95% CI
Stratum 1	Ref	-	-	-
Stratum 2	0.93	0.09	0.46	(0.77, 1.13)
Stratum 3	0.86	0.09	0.13	(0.70, 1.04)
Stratum 4	0.93	0.10	0.53	(0.75, 1.16)
Stratum 5	0.86	0.09	0.16	(0.70, 1.06)
Stratum 6	0.73	0.09	0.009	(0.58, 0.93)
Stratum 7	0.66	0.08	0.001	(0.52, 0.85)
Stratum 8	0.75	0.09	0.02	(0.58, 0.96)
Baseline home-level resident incidence	1.02	0.01	0.07	(1.00, 1.04)
Agency 1	0.77	0.07	0.004	(0.65, 0.92)
Agency 2	0.49	0.05	< 0.001	(0.41, 0.59)
Agency 3	1.14	0.11	0.17	(0.94, 1.37)
Agency 4	1.16	0.11	0.13	(0.96, 1.40)
Agency 5	0.91	0.09	0.37	(0.75, 1.11)
Agency 6	Ref	-	-	-
Bedrooms	1.00	0.01	0.65	(0.98, 1.03)
Home intensity	1.04	0.07	0.52	(0.92, 1.18)
Staff-to-resident ratio	0.96	0.02	0.06	(0.92, 1.00)
Prevalence of vaccinated residents	1.00	0.00	0.34	(1.00, 1.00)
Prevalence of vaccinated vax staff	1.00	0.00	0.54	(1.00, 1.00)
Prevalence of prior resident infection	1.00	0.00	0.02	(1.00, 1.00)
Prevalence of prior staff infection	1.00	0.00	0.04	(0.99, 1.00)

Mean age of resident	1.00	0.00	0.48	(0.99, 1.00)
Mean age of staff	0.99	0.01	0.12	(0.98, 1.00)
Proportion of non-white residents	1.00	0.00	0.75	(1.00, 1.00)
Proportion of non-white staff	1.00	0.00	0.21	(1.00, 1.00)
Proportion of male residents	1.00	0.00	0.87	(1.00, 1.00)
Proportion of male staff	1.00	0.00	0.89	(1.00, 1.00)
City-level incidence rate	1.04	0.04	0.22	(0.98, 1.11)
Linear time trend	4.11	0.34	< 0.001	(3.49, 4.84)
Quadratic time trend (time^2)	0.94	0.00	< 0.001	(0.93, 0.95)
Intervention arm main effect	0.39	0.26	0.16	(0.11, 1.45)
Intervention x time interaction	1.18	0.15	0.17	(0.93, 1.51)
Intervention x time^2 interaction	0.99	0.01	0.19	(0.98, 1.00)
Variance component	0.00	0.00		(0.00, 0.00)

*Staff to resident ratio is the number of unique staff who worked in the home during baseline divided by the number of resident beds in the home. Prevalence of vaccinated individuals and prior infection, mean age, proportion of non-white individuals and city-level incidence rate per 100 people were calculated at baseline.
